# Sustained Exocytosis after Action Potential-Like Stimulation at Low Frequencies in Mouse Chromaffin Cells Depends on a Dynamin-Dependent Fast Endocytotic Process

**DOI:** 10.3389/fncel.2016.00184

**Published:** 2016-07-26

**Authors:** José Moya-Díaz, Yanina D. Álvarez, Mauricio Montenegro, Lucas Bayonés, Ana V. Belingheri, Arlek M. González-Jamett, Ana M. Cárdenas, Fernando D. Marengo

**Affiliations:** ^1^Departamento de Fisiología y Biología Molecular y Celular, Facultad de Ciencias Exactas y Naturales, Instituto de Fisiología, Biología Molecular y Neurociencias, Universidad de Buenos Aires, Consejo Nacional de Investigaciones Científicas y TécnicasBuenos Aires, Argentina; ^2^Centro Interdisciplinario de Neurociencia de Valparaíso, Facultad de Ciencias, Universidad de ValparaísoValparaíso, Chile

**Keywords:** membrane capacitance, endocytosis, secretion, Ca^2+^ current, immediately releasable pool, dynamin

## Abstract

Under basal conditions the action potential firing rate of adrenal chromaffin cells is lower than 0.5 Hz. The maintenance of the secretory response at such frequencies requires a continuous replenishment of releasable vesicles. However, the mechanism that allows such vesicle replenishment remains unclear. Here, using membrane capacitance measurements on mouse chromaffin cells, we studied the mechanism of replenishment of a group of vesicles released by a single action potential-like stimulus (AP_ls_). The exocytosis triggered by AP_ls_ (ETAP) represents a fraction (40%) of the immediately releasable pool, a group of vesicles highly coupled to voltage dependent calcium channels. ETAP was replenished with a time constant of 0.73 ± 0.11 s, fast enough to maintain synchronous exocytosis at 0.2–0.5 Hz stimulation. Regarding the mechanism involved in rapid ETAP replenishment, we found that it depends on the ready releasable pool; indeed depletion of this vesicle pool significantly delays ETAP replenishment. On the other hand, ETAP replenishment also correlates with a dynamin-dependent fast endocytosis process (τ = 0.53 ± 0.01 s). In this regard, disruption of dynamin function markedly inhibits the fast endocytosis and delays ETAP replenishment, but also significantly decreases the synchronous exocytosis during repetitive AP_ls_ stimulation at low frequencies (0.2 and 0.5 Hz). Considering these findings, we propose a model in where both the transfer of vesicles from ready releasable pool and fast endocytosis allow rapid ETAP replenishment during low stimulation frequencies.

## Introduction

Under basal conditions the action potential firing rate of chromaffin cells is low, approximately 0.2–0.5 Hz, while under stress the system approaches 10–20 Hz (Brandt et al., 1976; Kidokoro and Ritchie, 1980; Holman et al., 1994; Chan and Smith, 2001). The maintenance of secretion in such range of frequencies requires the continuous refilling of releasable pools of vesicles (Smith et al., 1998; Sorensen, 2004) at rates that match the exocytotic activity. Therefore, it is important to define the mechanisms that contribute to the refilling of these pools.

It is well known that intense stimulation of chromaffin cells provokes an important global calcium (Ca^2+^) increase (Marengo and Monck, 2003), promoting in turn the substantial exocytosis of vesicles that are at variable location respect to voltage dependent Ca^2+^ channels (VDCC), with predominance of asynchronous over synchronous exocytosis (Chow et al., 1992; Zhou and Misler, 1995). The replenishment of these vesicles is mainly produced by mobilization from upstream to downstream pools (Voets et al., 1999). On the other hand, it has been shown by several investigators that single brief stimulation, which approximates to action potential duration, induces the synchronous release of vesicles closely coupled to VDCC (Horrigan and Bookman, 1994; Moser and Neher, 1997b; Voets et al., 1999) from a pool that was defined as the immediately releasable pool (IRP). Furthermore, action potential waveform stimuli applied at low frequencies (0.5 Hz) on chromaffin cells reportedly induce the release of catecholamines through Ω-shape kiss-and-run fusion events (Fulop et al., 2005), a process that probably allows fast local recycling of vesicles (Harata et al., 2006). However, the mechanisms involved in vesicle replenishment during low stimulation frequencies remain unknown. In this work, we investigated the possible mechanisms of vesicle replenishment that operate at low stimulation frequencies. Because, it is expected that action potentials applied at low frequencies would induce the exocytosis of vesicles tightly coupled to VDCC (Neher, 1998; Oré and Artalejo, 2005), we particularly analyzed the replenishment of vesicles included in the IRP.

As aforementioned, the IRP is a group of ready releasable vesicles sensitive to short depolarizations because of their proximity to VDCC (Horrigan and Bookman, 1994; Voets et al., 1999; Marengo, 2005; Alvarez and Marengo, 2011). The sustained participation of IRP in chromaffin cell exocytosis during repetitive action potential stimulation depends on the refilling rate of this pool. A previous study indicates that the refilling of IRP is very slow (Moser and Neher, 1997b), making difficult the participation of this pool even at low stimulation frequencies. In this work, we analyzed this subject by comparing the refilling of IRP after total depletion versus the replenishment of the group of vesicles released by a stimulus that approximates the shape of a chromaffin cell action potential (AP_ls_) (Chan et al., 2005b). While the whole IRP was refilled with a time constant of 7.5 s, the small group of vesicles released by AP_ls_ (ETAP) was replenished with a time constant of 0.7–1 s. This fast replenishment could account for the maintenance of synchronous exocytosis during repetitive stimulation at frequencies below 0.5 Hz. Finally, our results support the idea that the rapid ETAP replenishment relies in both the transfer of vesicles from the RRP and a dynamin-dependent fast endocytosis mechanism.

## Materials and Methods

### Mouse Adrenal Chromaffin Cell Preparation

All animal procedures were performed under protocols approved by the Consejo Nacional de Investigaciones Científicas y Técnicas (Argentina), and are in accordance with the National Institute of Health Guide for the Care and Use of Laboratory Animals (NIH publication 80-23/96), USA, and local regulations.

Adrenal glands from two 13–18 days old female/male 129/sv mice were used in each culture. Chromaffin cells were isolated and cultured following the procedures described by Perez Bay et al. (2012). Briefly, mechanically isolated adrenal medullas were digested for 25 min in Hanks with papain (0.5–1 mg/ml) at 37°C, and disrupted with a yellow tip in Dulbecco’s modified Eagle’s medium low glucose, supplemented with 5% fetal calf serum, 5 μl/ml penicillin/estreptomicina, 1.3 μl/ml gentamicin, 1 mg/ml bovine seroalbumin, and 10 μM citosine-1-β-D-arabinofuranoside. The cell suspension was filtered through 200 and 50 μm pore meshes, and cultured on poly-L-lysine pretreated coverslips at 37°C, 95% O_2_ – 5% CO_2_.

### Whole Cell Patch-Clamp and Membrane Capacitance Measurements

The patch-clamp set up comprised an inverted microscope (Olympus IX71, Olympus, Japan), a patch-clamp amplifier (EPC10 double patch clamp amplifier, Heka Elektronik, Lambrecht, Germany) and a personal computer. The application of the stimulation protocols and the data acquisition were controlled by the Patchmaster software (Heka, Lambrecht, Germany). Chromaffin cells were washed in extracellular solution composed of (mM) 120 NaCl, 20 Hepes, 4 MgCl_2_, 5 CaCl_2_, 5 mg/ml glucose and 1 μM tetrodotoxin (pH 7.3), and mounted on an inverted microscope (Olympus IX71). The standard internal solution used in the patch-clamp pipettes (3–5 MΩ) contained (in mM) 95 Cs d-glutamate, 23 Hepes, 30 CsCl, 8 NaCl, 1 MgCl_2_, 2 Mg-ATP, 0.3 Li-GTP, and 0.5 Cs- EGTA (pH 7.2). These solutions were designed to selectively measure voltage dependent Ca^2+^ currents (I_Ca2+_), and to maximize the exocytosis of vesicles tightly coupled to voltage-dependent Ca^2+^ channels (Alvarez et al., 2008). We have used these solutions previously to study the exocytosis of IRP (Marengo, 2005; Alvarez et al., 2008, 2013). In order to mimic a more physiological condition, in another group of experiments, we reduced the extracellular Ca^2+^ to 1.8 mM and the intracellular EGTA to 70 μM. The holding potentials were not corrected for junction potentials. We considered that the recorded cells were “leaky,” and discarded, when the leak current measured at the holding potential of -80 mV was bigger than -30 pA. We also discarded cells with series resistance bigger than 12 MΩ. The average series resistance in our control experiments was 9.7 ± 0.3 MΩ (*n* = 35). The cell C_m_ was determined by the sine + dc method (Gillis, 1995) implemented through the lock-in extension of Patchmaster. The command voltage applied to the cell was composed of the sum of a sinusoidal voltage (800 Hz, 60 mV peak to peak) and a holding potential of -80 mV. The data were filtered at 3 kHz. Patch pipettes were coated with dental wax to minimize their stray capacitance and to achieve a better C-fast compensation. The experiments were carried out at room temperature (22–24°C).

### Experimental Protocols

We used the voltage-clamp mode of patch-clamp/whole-cell configuration. To induce the exocytosis of IRP, we employed single 50 ms squared depolarizations from the holding potential of -80 to +10 mV. We also stimulated the cells with AP_ls_ composed by a 2.5 ms ascendant voltage ramp from -80 to +50 mV, followed by a 2.5 ms descendant ramp that returns to holding potential.

To study the replenishment of IRP or ETAP, we applied a pair of identical stimuli (squared 50 ms depolarizations or AP_ls_, respectively) separated by variable time periods, and expressed the degree of replenishment as the ratio between the exocytosis evoked by the second stimulus (C_m2_) and the exocytosis evoked by the first stimulus (C_m1_). The ratio C_m2_/C_m1_ was then plotted against the period between first and second stimuli.

The concentration of the anti-dynamin (anti-Dyn) or anti-GFP antibody in the internal solution was 7 nM, or 14 nM when was indicated. We wait a minimum of 5 min after whole cell establishment before to start the measurements, to allow the diffusion of antibodies to cytosol. Considering a series resistance of 9.7 and MW = 150 kD, anti-Dyn would reach in this period a 63% of steady state final cytosolic concentration, what was enough to block completely endocytosis (see **Figure 8**). Similarly, the GST fusion protein GST-Dyn_829-842_ (MW: 27.7 kD; see below) would reach approximately 82% of final concentration (Pusch and Neher, 1988). The effectiveness of anti-Dyn in inhibiting endocytosis was previously validated in mouse chromaffin cells microinjected with 7 nM of the antibody and tested for internalization of a transferrin-conjugate and an anti-dopamine-β hydroxylase antibody (Supplementary Figure S1).

### Materials

Bovine serum albumin, poly-L-lysine, cytosine-1-β-D-arabinofuranoside, and papain were obtained from Sigma (St. Louis, MO, USA); Dulbecco’s modified Eagle’s medium, fetal calf serum, gentamicin and penicillin/streptomicin from Gibco (Carlsbad, CA, USA); tetrodotoxin from Alomone Labs (Jerusalem, Israel); the monoclonal antibody against dynamin (it binds dynamin 1 and 2) from BD Biosciences (San Jose, CA, USA). The GST fusion protein GST-Dyn_829-842_, which contains the amino-acids 829 to 842 of human dynamin (PPQVPSRPNRAPPG) was obtained as described before (Gonzalez-Gutierrez et al., 2007). As a negative control of GST-Dyn_829-842_, we used a GST fusion protein containing mutations at positions 835 (arginine to aspartic acid) and 836 (proline to alanine), which reportedly does not disrupt the binding to the amphiphysin SH3 domain (Grabs et al., 1997).

### Data Analysis and Statistics

In some cells, immediately after the application of a depolarization pulse, we noted the presence of a brief transient capacitance change, probably associated to sodium channels gating (Horrigan and Bookman, 1994; Chow et al., 1996). This transient became negligible 50 ms after the end of depolarization (exponential time constant = 14 ± 2 ms; 14 measurements in 9 cells; for examples see Supplementary Figure S2). A capacitive artifact with similar kinetics was reported before in bovine and embryonic mouse chromaffin cells (Chow et al., 1996; Gong et al., 2005). To avoid any influence of this fast capacitance transient, we measured the capacitance increase caused by each stimulus as the difference between the mean capacitance measured in a 100 ms window starting 60 ms after the end of the depolarization minus the mean pre-stimulus capacitance also measured in a 100 ms window.

Data are expressed as mean values ± standard error. We used a Student’s “*t*” test for comparisons between two groups of independent data samples. One way ANOVA was used for multiple independent data samples, and Bonferroni test for comparisons between groups. If samples did not pass normality test we used Kruskal–Wallis one way analysis of variance on ranks. To fit the replenishment curves, we used the non-linear curve-fitting option in Origin (Microcal Software).

## Results

### Replenishment of IRP after Depletion

The exocytosis of IRP was induced by application of a 50 ms square depolarization pulse (from -80 to +10 mV). The change in whole cell C_m_ (ΔC_m_) induced by this stimulus was synchronous with the depolarization pulse, and averaged 27 ± 2 fF (*n* = 53). We previously demonstrated that this stimulus induces the complete release of IRP in our chromaffin cell preparation (Alvarez et al., 2013). Moreover, the application of this stimulus in the presence of 15 mM Ca^2+^ in the external solution increased the I_Ca2+_ in approximately 60%, but the exocytosis of IRP was not affected (25 ± 2, *n* = 9). This estimation of IRP is similar to previous reports (Horrigan and Bookman, 1994; Voets et al., 1999; Alvarez et al., 2008; Alvarez and Marengo, 2011).

**FIGURE 1:**
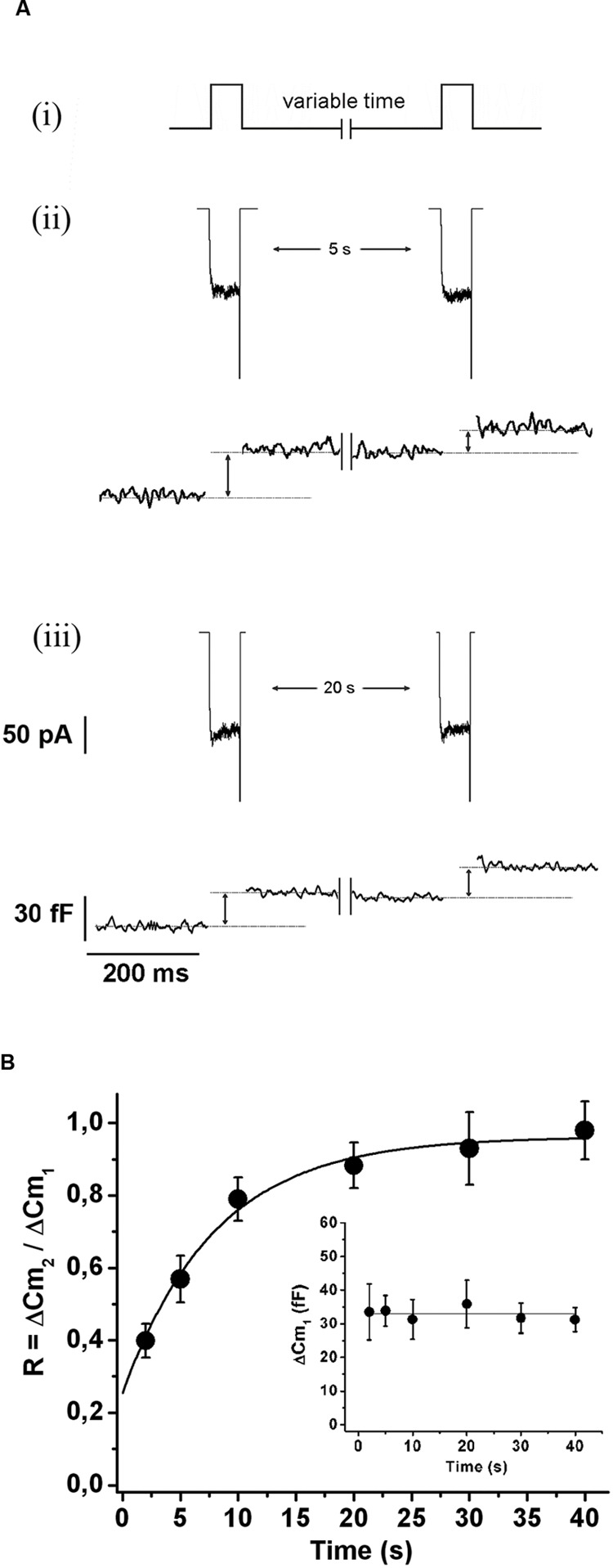
**Immediately releasable pool refilling after depletion. (A)** (i) Scheme of the stimulation protocol. In order to determine the kinetics of IRP replenishment, a pair of 50 ms depolarizations (from -80 to +10 mV) with a variable time interval between them was applied. (ii) and (iii) I_Ca2+_ (top) and changes in cellular capacitance (bottom) obtained during typical experiments when 5 and 20 s intervals between stimuli were, respectively, applied. **(B)** The relative replenishment of IRP after depletion (expressed as ΔC_m2_/ΔC_m1_; where ΔC_m1_ and ΔC_m2_ represents the change in capacitance induced by the first and the second depolarization, respectively) was plotted against the time interval between 50 ms depolarizations. Each point represents the average of measurements obtained in at least 11 independent cells. The averaged values were fitted to a monoexponential growing function (R = R_0_ + A.(1 - e^
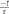
^)), obtaining a value at time zero *R*_0_ of 0.22 ± 0.05, an asymptote *A* of 0.73 ± 0.05, a time constant τ of 7.5 ± 1.1 s, and a correlation coefficient *R* > 0.9957. The graph in the inset represents the averaged changes in capacitance induced by the first depolarization of the paired pulse protocol described above, and the continuous line represents the general average for these measurements.

In order to evaluate the kinetics of IRP refilling after depletion, we first applied a 50 ms square depolarization pulse to deplete IRP, and after a variable period (between 2 and 40 s), we applied a second 50 ms pulse to assess the replenishment of the pool (**Figure 1Ai**). There was no difference between the amplitude of the I_Ca2+_ evoked by the first and the second depolarization pulse (157 ± 10 and 150 ± 11 pA, respectively; *n* = 109). **Figures 1Aii,iii** illustrate typical records of I_Ca2+_ and ΔC_m_ obtained by the application of this protocol with 5 and 20 s intervals, respectively. After 5 s, only a fraction of the ΔC_m_ associated to IRP was recovered, but 20 s was enough to recover almost completely the exocytosis of this pool. **Figure 1B** shows the average results of this type of experiments, where the relative replenishment (R) of IRP is represented by the ratio between the capacitance increase induced by the second pulse (ΔC_m2_) over the capacitance increase induced by the first pulse (ΔC_m1_). The exocytosis induced by the first pulse was similar among the different intervals tested (**Figure 1B**, inset). The experimental points of **Figure 1B** were fitted to a single exponential growing function (*R* > 0.9957), obtaining a time constant τ of 7.5 ± 1.1 s and an intercept value at time zero of 0.22 ± 0.05. These parameters are very similar to those previously reported (Moser and Neher, 1997b). This time constant predicts the replenishment of 90% of IRP in approximately 20 s.

### Replenishment of the Group of Vesicles Released by an AP_ls_

**FIGURE 2:**
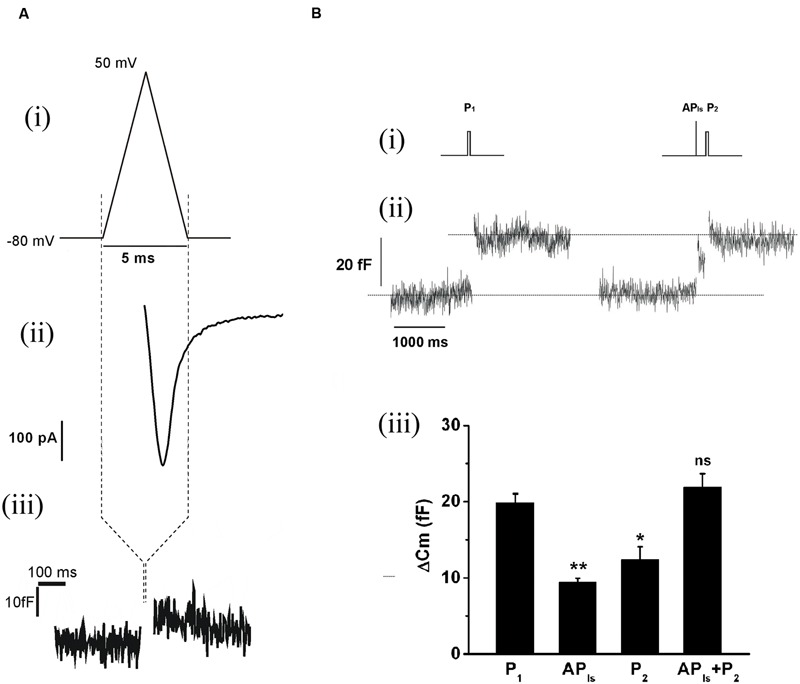
**The application of an AP_ls_ releases a subgroup of vesicles from IRP. (A)** (i) The AP_ls_ was composed by an ascending ramp lasting 2.5 ms from the holding potential (-80 mV) to +50 mV, and a descending ramp of equal duration that returns to the holding potential. (ii) and (iii) Examples of the I_Ca2+_ and the capacitance jump induced by this type of stimulus. The capacitance record is in a different time scale. **(B)** (i) Stimulation protocol consisting in the sequential application of a 50 ms square depolarization pulse (P_1_) to evaluate the size of IRP in control conditions, then after 20 s an AP_ls_ was applied, and almost immediately (200 ms) the cell was stimulated with another 50 ms pulse (P_2_) to evaluate the effect of the previous AP_ls_ on IRP size. (ii) Typical record and (iii) bar diagram of averaged results (*n* = 9) obtained from this type of experiments. AP_ls_+P_2_ represents the sum of the capacitance changes induced by AP_ls_ and P_2_, obtained in each experiment. The four conditions represented in the bar diagram were compared by ANOVA (*p* < 0.001), and AP_ls_, P_2_ and AP_ls_+P_2_ were compare against P_1_ (^∗^*p* < 0.003 and ^∗∗^*p* < 0.001) by Bonferroni test.

**FIGURE 3:**
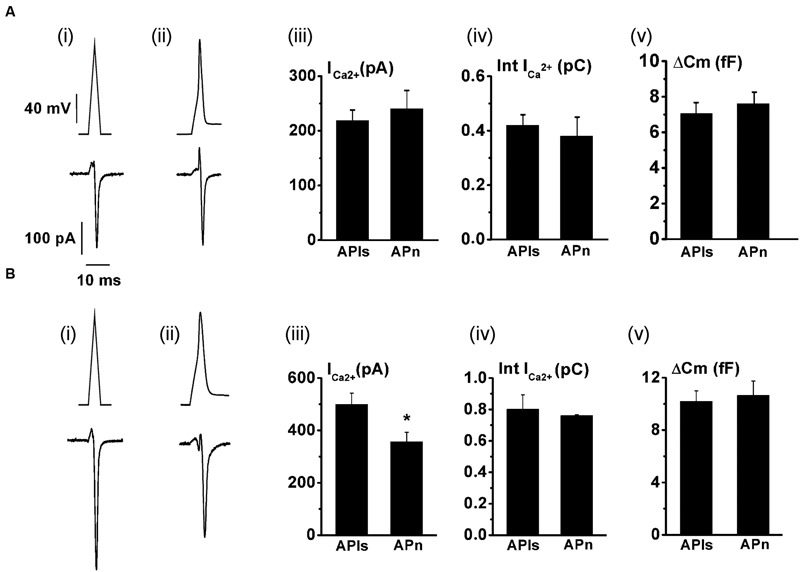
**The application of an AP_ls_ induces similar Ca^2+^ entry and exocytosis than a native action potential.** The Ca^2+^ current and the exocytosis induced by voltage-clamp application of previously obtained native action potentials (APn) (*n* = 10) were measured, and the results compared with independent measurements obtained in cells stimulated with AP_ls_ (*n* = 10) at either 1.8 mM external Ca^2+^/70 μM internal EGTA **(A)**, or at 5 mM external Ca^2+^/0.5 mM internal EGTA **(B)**. **(A)** (i) and **(B)** (i) represents AP_ls_ (top), while **(A)** (ii) and **(B)** (ii) represents the action potential templates obtained in current clamp experiments (top), and typical examples of Ca^2+^ current records (bottom) are represented for all the conditions. The bar graphs in (iii), (iv) and (v) summarizes the I_Ca2+_, the I_Ca2+_ integral (Int-Ica), and the ΔCm, respectively. ^∗^*p* < 0.05.

The replenishment of IRP, as represented in **Figure 1**, is too slow to sustain a significant participation of IRP during repetitive stimulation, even at low frequencies. On the other hand, it is important to note that chromaffin cell exocytosis in physiological conditions is induced by action potentials. A chromaffin cell action potential is much shorter than a square 50 ms pulse, and therefore it is expected to release a smaller amount of vesicles. To approximate the amplitude, shape and duration of a chromaffin cell action potential, we applied a voltage pulse composed by an ascending and a descending ramp (see methods and **Figure 2Ai**). The application of an AP_ls_ induced a I_Ca2+_ of 488 ± 34 pA (*n* = 35) and a capacitance jump of 11 ± 1 fF (*n* = 35) (see **Figures 2Aii,iii** for individual examples). We used this type of stimulus along the work because it allowed us to build in our software a variety of stimulation protocols, fixing precisely the frequency of stimuli, and maintaining their amplitude constant along paired or train simulation. However, since the shape of action potential may affect calcium current (Duan et al., 2003), we first compared the I_Ca2+_, the Ca^2+^ entry measured as the time integral of I_Ca2+_ (Int-Ica) and ΔCm induced by AP_ls_ with those induced by native action potentials. To obtain native action potentials, chromaffin cells were held in whole cell current clamp configuration and injected with squared current pulses of 120 pA and 2 ms duration. The resulting action potential (**Figures 3Aii,Bii**, top) was used as a stimulus template in voltage clamp experiments (Chan and Smith, 2001). These experiments were performed in 5 mM external Ca^2+^/0.5 mM internal EGTA, as well as in 1.8 mM external Ca^2+^/70 μM internal EGTA, then they were compared with separate sets of independent measurements obtained by application of AP_ls_. Int-Ica and ΔCm induced by native action potentials or APls were similar for both external Ca^2+^ concentrations (**Figures 3A,B**). However, the I_Ca2+_ induced by native action potentials at 5 mM external Ca^2+^/0.5 μM EGTA showed a small, but significant, reduction with respect to APls (**Figures 3Bi–iii**).

**FIGURE 4:**
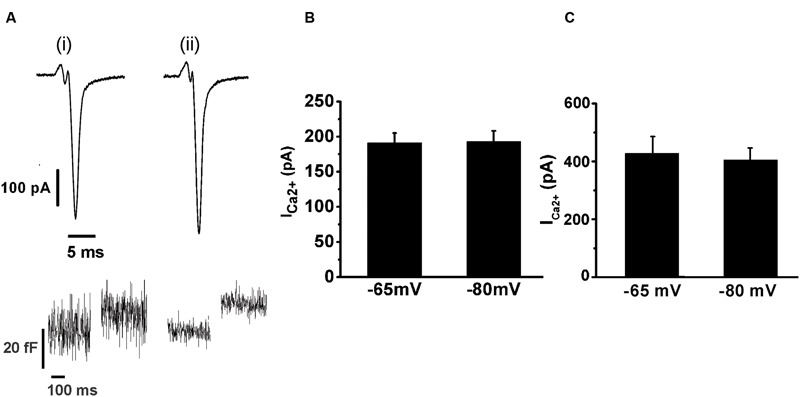
**The application of an AP_ls_ induces similar I_Ca2+_ at -65 or -80 mV holding potentials.** The I_Ca2+_ and the ΔC_m_ induced by AP_ls_ applied at -65 and -80 mV holding potentials were measured in paired experiments. **(A)** (i) and (ii) represents typical records of I_Ca2+_ (top) and ΔC_m_ (bottom) obtained at -65 and -80 mV holding potential, respectively, at 5 mM external Ca^2+^/0.5 mM internal EGTA. The bar graphs in **(B)** and **(C)** summarizes the I_Ca2__+_measurements for 1.8 mM external Ca^2+^/70 μM internal EGTA, and for 5 mM external Ca^2+^/0.5 mM internal EGTA, respectively (*n* = 10 for both conditions).

We also tested whether the holding potential of -80 mV might overestimate I_Ca2+_ and consequently exocytosis, because of the recruitment of additional VDCC. Thus, we performed paired measurements of I_Ca2+_ and ΔCm in response to the application of AP_ls_ at -80 mV plus ± 30 mV vs. a more physiologic holding (-65 mV) plus ± 15 mV sinusoidal voltage. The results, represented in **Figure 4**, show that there was no significant difference in I_Ca2+_ between both conditions. The exocytosis, reported by ΔCm, was too noisy for the experiments performed at -65 mV (see **Figure 4Ai**, bottom), probably because of the shorter amplitude of the sinusoidal voltage. Therefore, although our estimations of exocytosis were very similar between -65 and -80 mV holding potentials, we decided not include these data in the figure. Conversely, the measurements obtained at -80 mV holding potential, which uses ±30 mV sinusoidal voltage, had an acceptable signal/noise relationship (see **Figure 4Aii**, bottom). We must note in addition, that we also failed to find differences between I_Ca2+_, Int-Ica or ΔCm induced by native action potentials applied at -65 and -80 mV holding potentials (data not shown). Therefore, considering that the -80 mV holding potential and the application of AP_ls_ did not modified substantially the I_Ca2+_, Ca^2+^ entry and exocitosis in comparison with more physiological -65 mV and native action potentials, we used the first conditions to design our stimulation protocols along the work. Finally, the decrease of external Ca^2+^ from 5 to 1.8 mM significantly reduced (*p* < 0.05) the sizes of I_Ca2+_ and exocytosis (**Figures 3** and **4**). As AP_ls_ induces the exocytosis of a small amount of vesicles, the next experiments were performed with the higher Ca^2+^ concentration to improve our signal resolution. Thereafter, only when it is indicated, we did experiments using the more physiological 1.8 mM external Ca^2+^ and 70 μM internal EGTA.

Because AP_ls_ is a very short stimulus, it should release only vesicles from IRP. To evaluate this prediction, we sequentially applied a square 50 ms depolarization pulse (P_1_) to obtain a control estimation of IRP for each particular experiment, and 20 s later a single AP_ls_ followed almost immediately (200 ms) by a second 50 ms depolarization pulse (P_2_) (**Figure 2Bi**). As expected, the results show that AP_ls_ induced an exocytotic response markedly smaller than IRP. More important, the second 50 ms pulse (P_2_) also triggered a significantly smaller response than control IRP estimation (*p* < 0.003), and the addition of the exocytosis induced by both the AP_ls_ and the contiguous 50 ms pulse (AP_ls_+P_2_) gives a value almost identical to control IRP (**Figures 2Bii,iii**). These results indicate that the group of vesicles released by a single AP_ls_ (ETAP) is a fraction of that we define as IRP.

**FIGURE 5:**
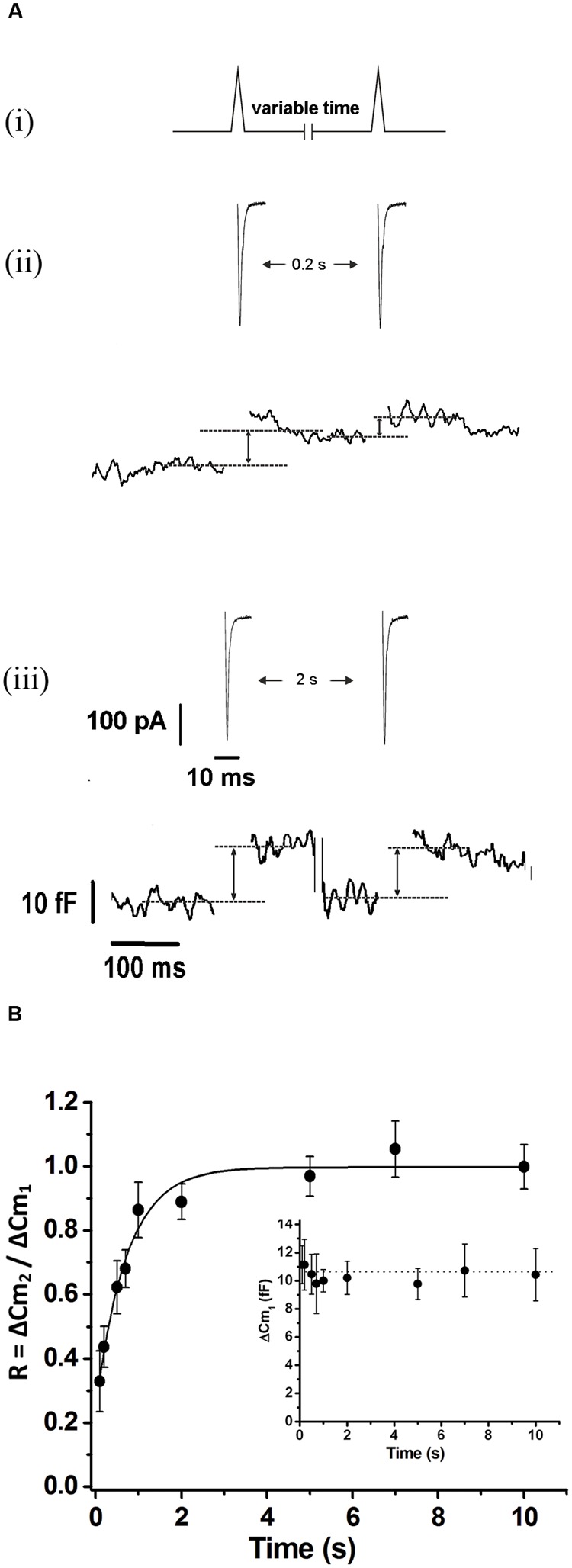
**Exocytosis triggered by action potential like stimulus replenishment after depletion. (A)** (i) Scheme of the stimulation protocol. We applied two AP_ls_ separated by a variable time interval to evaluate the ETAP replenishment. (ii) and (iii) I_Ca2+_ (top) and changes in cellular capacitance (bottom) obtained during typical experiments where 0.2 and 2 s intervals between stimuli were, respectively, applied. Capacitance records are shown in a different time scale. **(B)** The relative replenishment of ETAP (expressed as ΔC_m2_/ΔC_m1_; where ΔC_m1_ and ΔC_m2_ represents the change in capacitance induced by the first and the second AP_ls_, respectively) was plotted against the time interval between AP_ls_. Each point represents the average of measurements obtained in at least 11 independent cells. The averaged values were fitted to a monoexponential growing function (R = R_0_ + A.(1 - e^
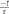
^)), obtaining a value at time zero R_0_ of 0.24 ± 0.05, an asymptote A of 0.75 ± 0.05, a time constant τ of 0.73 ± 0.11 s, and a correlation coefficient *R* > 0.9886. The graph in the inset represents the averaged changes in capacitance induced by the first AP_ls_ of the paired pulse protocol described above, and the dotted line represents the general average for these measurements.

To study the replenishment of ETAP, we applied the protocol represented in **Figure 5Ai**. Basically, it consists in a pair of AP_ls_ separated by a variable period, between 0.2 and 10 s. **Figures 5Aii,iii** illustrate examples of the measurements obtained by the application of this protocol for 0.2 and 2 s intervals, respectively. The **Figure 5B** summarizes the results obtained from this type of experiments. The relative replenishment R was defined by the ratio between the exocytosis induced by the second AP_ls_ over the exocytosis induced by the first AP_ls_. The experimental points were well fitted to a single exponential function, showing an average time constant of 0.73 ± 0.1 s, which is ten times faster than that observed for the total IRP refilling (*p* < 0.01). In experiments performed with more prolonged intervals between stimuli (20, 30, and 40 s) (Supplementary Figure S3), the final plateau remained stable, so the fast replenishment kinetics of ETAP is not the consequence of a transitory overfilling process as was described previously for RRP (Smith et al., 1998).

**FIGURE 6:**
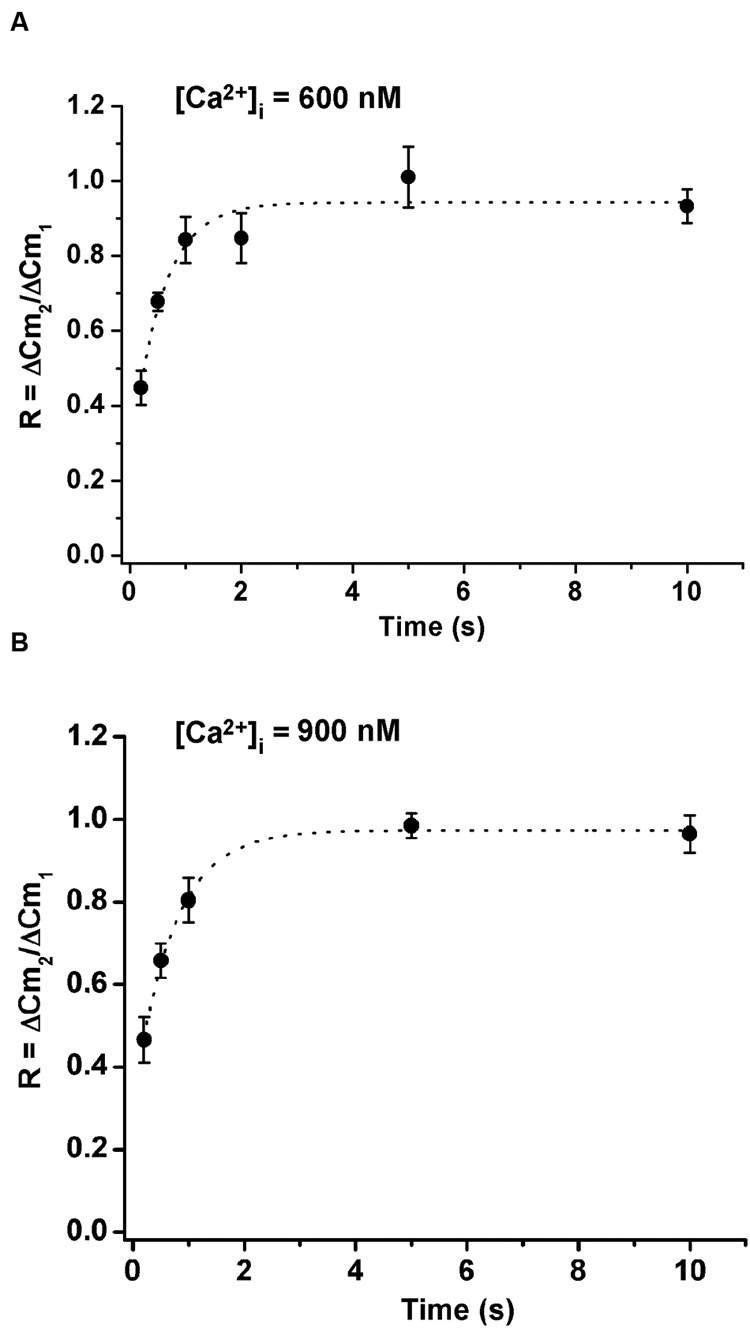
**Replenishment of ETAP after increasing cytosolic Ca^2+^ concentration by Ca^2+^ dialysis from the patch pipette.** The cytosolic Ca^2+^ concentration was increased by addition of 383 and 417 μM of Ca^2+^ to the internal solution, what results in approximately 600 nM **(A)** and 900 nM **(B)** free Ca^2+^ concentrations, respectively. In these experiments the extracellular Ca^2+^ concentration was kept at 5 mM. The averaged values were fitted to a monoexponential growing function (R = R_0_ + A.(1 - e^
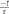
^)). In **(A)** the fitting parameters were, *R*o = 0.24 ± 0.14, *A* = 0.69 ± 0.14, τ = 0.55 ± 0.19 s, and *R* > 0.9722; and in **(B)** they were, *R*o = 0.30 ± 0.03, *A* = 0.67 ± 0.03, τ = 0.70 ± 0.06 s, and *R* > 0.9975. Each point represents the average of measurements obtained in at least 7 independent cells.

We also studied the influence of the cytosolic Ca^+2^ concentrations on ETAP replenishment. Therefore, we increased the cytosolic Ca^2+^ concentration by adding 383 and 417 μM of Ca^2+^ to the internal solution. Considering that the internal solution already has 0.5 and 2 mM ATP, the cytosolic free Ca^2+^ concentration approximately increased to 600 and 900 nM, respectively. We found that the increase of cytosolic Ca^2+^ did not significantly change ETAP replenishment (**Figures 6A,B**) in comparison with the experiments where Ca^2+^ was not added to the internal solution (**Figure 5B**).

### ETAP Replenishment Is Dependent on RRP Vesicle Content

**FIGURE 7:**
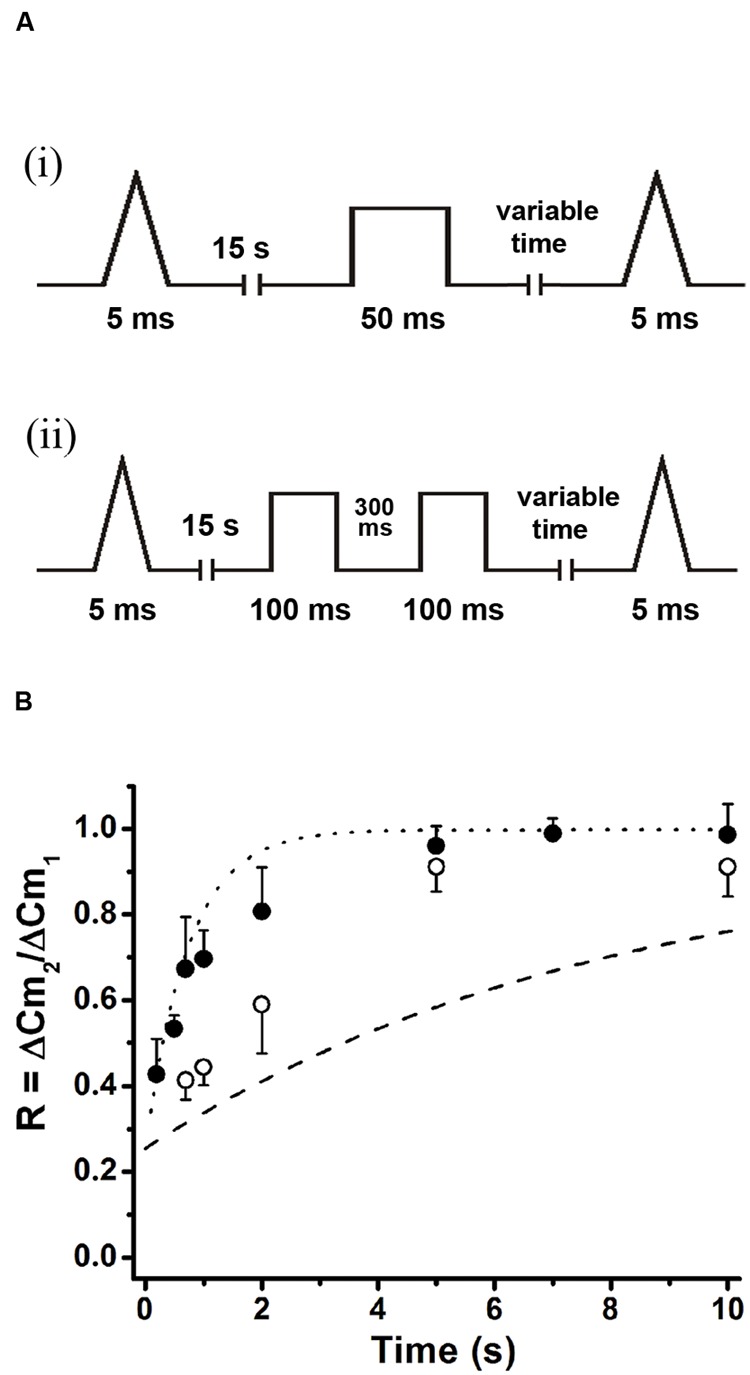
**ETAP replenishment depends on RRP vesicle content. (A)** Scheme of stimulation protocols. First, an AP_ls_ was applied to evaluate ETAP at control condition; then after 15 s a 50 ms square depolarization pulse was applied to deplete IRP (i), or a pair of 100 ms pulses separated by 300 ms were applied to deplete RRP (ii); and finally a second AP_ls_ was applied after a variable time interval to evaluate the replenishment of ETAP. **(B)** Summary of the results obtained in both types of experiments. The ETAP replenishment after IRP depletion is represented by filled circles (each point represents the average of at least six independent cells), and after RRP depletion is represented by open circles (each point represents the average of at least five independent cells). For comparison, we plotted the control ETAP replenishment taken from **Figure 5B** (dotted line) and the replenishment of IRP taken from **Figure 1B** (dashed line). The statistics of these experiments are represented in **Table 1**.

Classical work in chromaffin cells, as well as in other preparations, has described the refilling of releasable pools by transfer of vesicles through different pools organized in series (von Gersdorff and Matthews, 1997; Smith et al., 1998; Voets et al., 1999). On the other hand, it was also proposed that AP_ls_ applied at low frequencies in chromaffin cells induce a type of kiss-and-run exocytotic/endocytotic process (Fulop et al., 2005), in where the vesicles can be locally recycled. In order to determine which one of these mechanisms operate under the rapid ETAP replenishment, we first depleted vesicles pools that are expected to be located upstream of ETAP. Because ETAP is a subgroup of vesicles included in IRP (**Figure 2B**), it is possible that the rest of vesicles of IRP feeds ETAP in a sequential way. Therefore, we depleted IRP as a first intent to interfere with ETAP replenishment. **Figure 7Ai** shows the protocol used for this type of experiment. First, a single AP_ls_ was applied to have a control value for ETAP, then after 15 s the depletion of IRP was induced by a 50 ms depolarization pulse (ΔCm = 24 ± 5 fF, *n* = 7), and finally the replenishment of ETAP was tested by application of a second AP_ls_ after variable periods. The replenishment of ETAP under this experimental condition is represented in **Figure 7B** by the filled circles. For comparison, we plotted in the same graph the normal ETAP replenishment taken from **Figure 5B** (dotted line) and the first part of the normal IRP replenishment taken from **Figure 1B** (dashed line). It is clear that when IRP was depleted, the replenishment of ETAP still followed a fast kinetics. This result indicates that ETAP is replenished by a mechanism independent of IRP vesicle content.

Alternatively, ETAP might be replenished directly from RRP. To test this possibility, we depleted the RRP using the protocol represented in **Figure 7Aii** (Voets et al., 1999), and then analyzed how this protocol affects the ETAP replenishment. The double 100 ms pulse provoked capacitance increases of 42 ± 5 and 21 ± 3 fF (*n* = 6), respectively. The replenishment of ETAP (**Figure 7B**) under this experimental condition is represented by the opened circles. The shortest time intervals (0.2 and 0.5 s) were not considered because the double 100 ms depolarization pulse produced a significant inactivation of I_ca2+_ that persisted for 500 ms. In this condition, the relative values of replenishment after 0.7, 1, and 2 s intervals were significantly decreased in comparison with control ETAP replenishment (dotted line, see **Table 1** for statistical analysis). These results indicate that ETAP replenishment depends on the mobilization of vesicles from RRP.

**Table 1:** Summary of ETAP Recovery.

Time (s)	CONTROL	RRPdplt	RnDn	AntiDyn	RRPdplt+AntiDyn	GST-Dyn
0.2	0.44 ± 0.07 (11)	-	0.39 ± 0.03 (14)	0.41 ± 0.07 (10)	-	0.45 ± 0.06 (12)
0.5	0.62 ± 0.08 (13)	-	0.52 ± 0.03 (12)	0.61 ± 0.07 (15)	-	0.52 ± 0.05 (15)
0.7	0.68 ± 0.06 (13)	0.41 ± 0.04 (7)^∗∗^	0.52 ± 0.04 (10)	0.60 ± 0.07 (13)	0.40 ± 0.05 (12)#	
1	0.86 ± 0.08 (13)	0.44 ± 0.04 (5)^∗∗∗^	0.61 ± 0.03 (11)^∗^	0.64 ± 0.07 (15)^∗^	0.51 ± 0.04 (9)^∗∗∗^	0.62 ± 0.04 (16)^∗∗^
2	0.89 ± 0.05 (17)	0.59 ± 0.11 (7)^∗∗^	0.72 ± 0.03 (11)	0.59 ± 0.07 (15)^∗∗∗^	0.45 ± 0.07 (9)#	0.67 ± 0.05 (13)^∗^
5	0.97 ± 0.06 (15)	0.91 ± 0.07 (6)	1.03 ± 0.06 (15)	0.72 ± 0.06 (15)^∗∗^	0.47 ± 0.06 (9)#	0.73 ± 0.05 (14)^∗^
10	1.0 ± 0.07 (13)	0.91 ± 0.07 (7)	0.98 ± 0.06 (15)	0.82 ± 0.09 (15)	0.83 ± 0.05 (9)	0.86 ± 0.05 (15)

*Relative ETAP replenishment (expressed as ΔC_*m2*_/ΔC_*m1*_; where ΔC_*m1*_ and ΔC_*m2*_ represents the change in capacitance induced by the first and the second AP_ls_, respectively) at the different conditions studied in this work. Comparisons between different treatments at every particular time was performed by one way ANOVA, and if statistical differences were significant (*p* < 0.05) the comparisons between control and individual treatments were performed by Bonferroni (^∗^*p* < 0.05; ^∗∗^*p* < 0.02; ^∗∗∗^*p* < 0.005; ^#^*p* < 0.001). The number of measurements for each experimental situation is represented between parentheses. RRPdplt, depletion of RRP; RnDn, rundown of endocytosis; AntiDyn, anti-dynamin monoclonal antibody; GST-Dyn, GST-Dyn_*829-842*_ peptide.*

### ETAP Replenishment Correlates with a Fast Endocytotic Process

**FIGURE 8:**
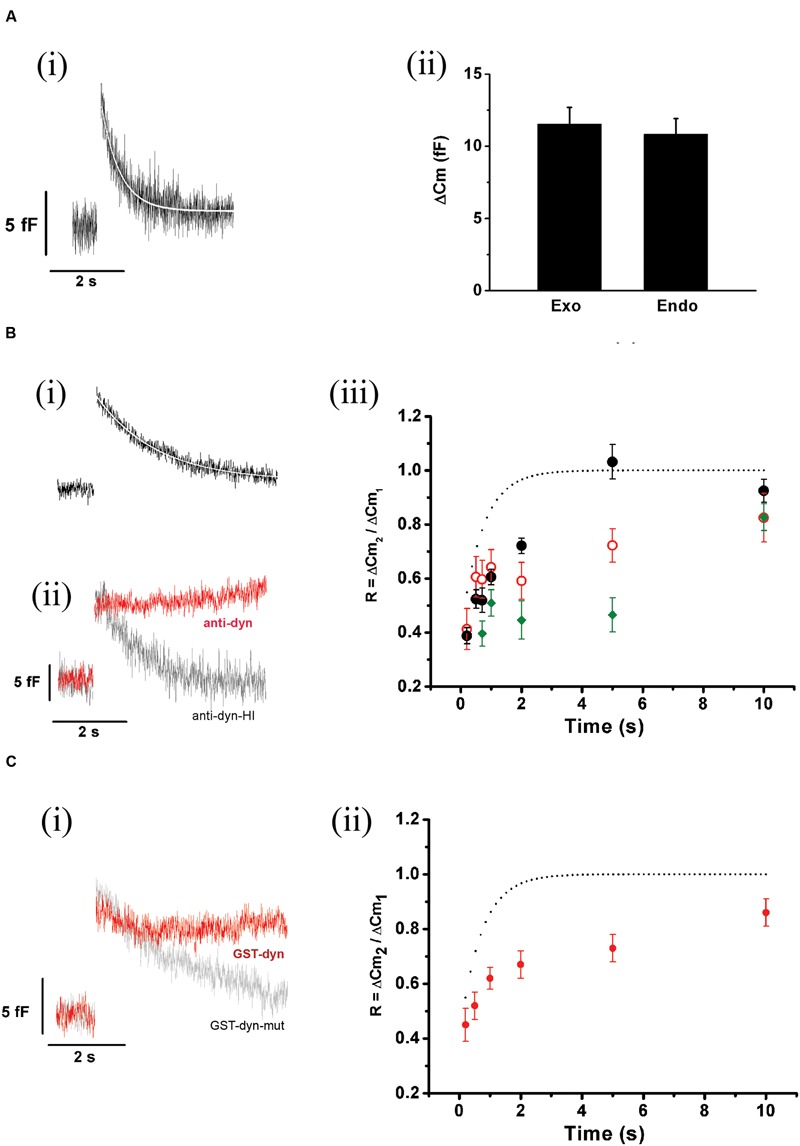
**ETAP replenishment depends on a rapid endocytosis process. (A)** (i) After ETAP, the membrane surface is recovered by a fast endocytosis. The figure represents the average of 35 records obtained in independent cells. The decay in the C_m_ was fitted to a single exponential equation (τ = 0.53 ± 0.01 s; *R* > 0.9446). (ii) The bar diagram represents the amplitudes of the exocytosis (Exo) and the following endocytosis (Endo) estimated from these experiments (*n* = 35). The ΔC_m_ values associated to these processes were no significantly different. **(B)** (i) This figure represents the average of the endocytosis records measured in 13 individual cells where the measurements started at least 5 min after whole cell establishment. The decay in C_m_ was fitted to a single exponential equation (τ = 1.65 ± 0.02 s, *R* = 0.9883). (ii) Average (in red) of 11 records of endocytosis obtained in an identical number of cells where a monoclonal antibody (7 nM) against dynamin (anti-dyn) was dialyzed for at least 5 min from the patch pipette. The average (in light gray) of five cells treated with a previously heat-inactivated antibody (anti-dyn-HI) is also represented. (iii) Replenishment of ETAP in three experimental conditions: when measurements were initiated 5 min after whole cell establishment (black filled circles), when an anti-dynamin antibody was added to the internal solution (red open circles), or when the anti-dynamin antibody was added to the internal solution and RRP was depleted by the protocol represented in **Figure 7A** (ii) (green diamonds). For comparison, the fitted curve obtained in control conditions (taken from **Figure 5B**) is shown as dotted line. **(C)** (i) This figure represents the average (in red) of the endocytosis records measured in 11 individual cells where the peptide GST-Dyn_829-842_ (30 nM) was dialyzed for 5 min from the patch pipette. This endocytotic process did not compensate previous exocytosis (the exocytosis was 10.3 ± 0.9 fF while endocytosis measured at 4 s after the end of the stimulus was 2.43 ± 0.87 fF, *p* < 0.05). The gray line represents the average of six cells dialyzed with the mutated peptide GST-Dyn-mut. (ii) Replenishment of ETAP in presence of GST-Dyn_829-842_ (red circles). The dotted line represents the fitted curve obtained in control conditions. The statistics for ETAP replenishment for all the conditions represented in this figure are included in **Table 1**.

Exocytosis triggered by action potential like stimulus was regularly followed by a fast decay in capacitance (**Figure 8Ai**). The amplitude of this decay was almost identical to the preceding exocytosis (**Figure 8Aii**). A very similar process was described previously by Chan and Smith (2001, 2003), and was associated to fast compensatory endocytosis. The average decrease in C_m_ (*n* = 35) associated to this endocytosis was fitted to a single exponential function with a time constant of 0.53 ± 0.01 s (*R* > 0.9446, white line in **Figure 8Ai**). Since this endocytosis rate was similar to ETAP replenishment rate, it is possible to hypothesize that both processes might be linked.

To analyze whether ETAP replenishment kinetics might be rate-limited by vesicle endocytosis, we applied various experimental approaches to interfere with fast endocytosis in our cells. It was described that fast compensatory endocytosis runs down in approximately 4 min after the whole cell configuration is established (Smith and Neher, 1997). It is important to note that our results are clearly consistent with a compensatory type of endocytosis, and not with other types, like excess retrieval, that does not washout in whole cell configuration (Smith and Neher, 1997; Burgoyne, 1998). We decided to take advantage of this feature as a way to inhibit fast endocytosis. Hence, we waited 5 min after the establishment of the whole cell configuration and started the typical protocol (**Figure 5Ai**) to determine the kinetics of ETAP replenishment. This 5 min period did not affect ETAP exocytosis (11 ± 1 fF). **Figure 8Bi** represents the average endocytosis obtained from 13 cells under these experimental conditions, which was fitted to a single-exponential decay function (white line) with a time constant of 1.65 ± 0.02 s. This time constant is quite larger than control (*p* < 0.01), resulting in a moderate inhibition of fast endocytosis. Interestingly, ETAP replenishment in this condition was also moderately delayed respect to the control condition [black filled circles in **Figure 8Biii**, and RnDn (for rundown of endocytosis) column in **Table 1**]. To further study this mechanism, we inhibited the fast endocytosis using a mouse monoclonal antibody against dynamin (anti-Dyn) (Gonzalez-Jamett et al., 2010) (see “Materials and Methods,” “Experimental Protocols”). Anti-Dyn (7 nM) was dialyzed through the patch pipette during 5 min after whole cell establishment. The I_Ca2+_ induced by AP_ls_ in these conditions was 352 ± 23 pA (*n* = 14). In this condition, the fast endocytosis after ETAP was completely inhibited (**Figure 8Bii**, red line). Such inhibition was totally suppressed when the antibody was previously heat-inactivated at 95°C during 5 min (**Figure 8Bii**, gray line), or replaced by an anti-GFP antibody (see Supplementary Figure S4A). Importantly, the application of anti-Dyn induced a pronounced delay in ETAP replenishment (**Figure 8Biii**, red open circles; **Table 1**). No additional effect on ETAP replenishment was observed when the antibody concentration was incremented to 14 nM (Supplementary Figure S4B). Finally, to additionally confirm the contribution of dynamin-dependent fast endocytosis on ETAP replenishment, we dialyzed the cells through the patch pipette with a 14-oligomer peptide GST-Dyn_829-842_ (see “Materials” in “Materials and Methods” section) containing the recognition motif for SH3 domain-containing proteins in the dynamin-1 proline-rich domain. It was previously demonstrated that this peptide inhibits dynamin recruitment to endocytotic sites (Shupliakov et al., 1997), as well as the endocytosis under low frequency stimulation in chromaffin cells (Fulop et al., 2008). The I_Ca2+_ induced by AP_ls_ in these conditions was 335 ± 49 pA (*n* = 15). The application of 30 μM of GST-Dyn_829-842_ to the internal solution almost completely abolished the fast endocytosis (**Figure 8Ci**, red line), compared to control conditions (**Figure 8Ai**) and to the application of a mutated version of the peptide defective in binding the SH3 domain of amphiphysin (see “Materials and Methods”) (**Figure 8Ci**, gray line). Furthermore, the application of GST-Dyn_829-842_ also significantly delayed ETAP recovery (**Figure 8Cii**; **Table 1**).

The experiments presented in this section support that ETAP replenishment is preceded by a dynamin-dependent fast endocytotic mechanism that contribute to replenish vesicles in a short time after they fused to the plasma membrane. In addition, the experiments of the previous section indicated that ETAP replenishment is also affected by depletion of RRP. Consequently, ETAP replenishment seems to be dependent on two different mechanisms: a dynamin-dependent fast endocytosis and vesicle transference from upstream pools. Therefore, we analyzed the effect of blocking RRP replenishment and fast endocytosis together. We applied the protocol represented in **Figure 7Aii** after 5 min of dialysis with anti-Dyn. This treatment provoked a dramatic inhibition of ETAP replenishment (**Figure 8Biii**, green diamonds. For statistical comparison please see **Table 1**). It is necessary to mention here that, we decided to compare the values of replenishment at individual times by ANOVA instead to compare the time constants between different conditions because some experimental treatments (see green diamonds and red open circles in **Figure 8Biii**) suggest that ETAP replenishment did not really behave as a single exponential process.

**FIGURE 9:**
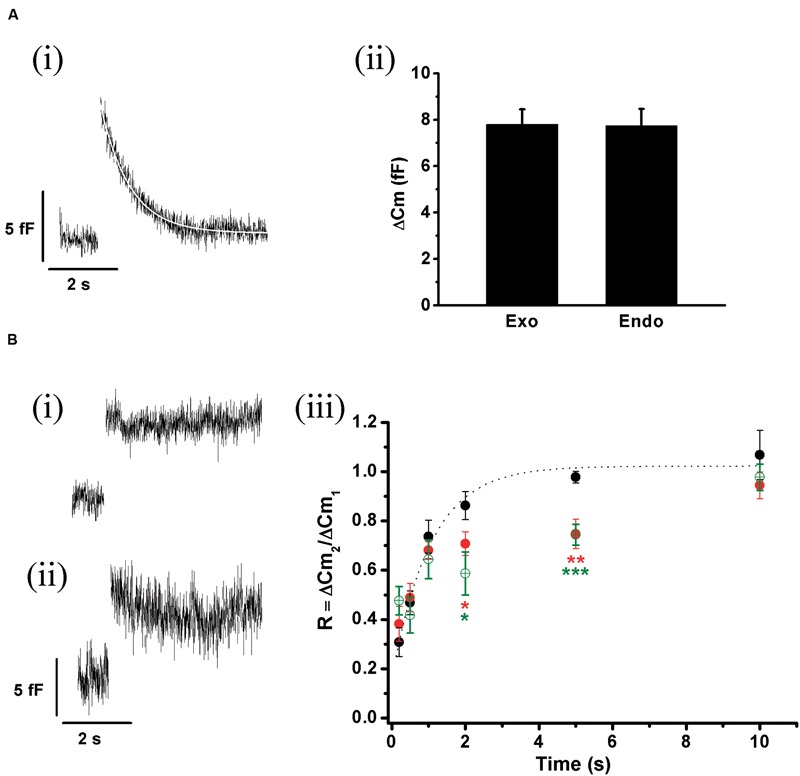
**ETAP replenishment at physiological Ca^2+^. (A)** The experiments of **Figure 5** were repeated using 1.8 mM external Ca^2+^/70 μM internal EGTA, which better represents physiological extracellular Ca^2+^ and cytosolic buffer capacity. (i) The figure represents the average of 14 records obtained in independent cells. The decay in the C_m_ was fitted to a single exponential equation (τ = 0.77 ± 0.06 s; *R* > 0.9783). (ii) The bar diagram represents the amplitudes of the exocytosis (Exo) and the following endocytosis (Endo) estimated from these experiments, which are not significantly different. **(B)** (i) and (ii) Average of representative endocytosis records obtained after the dialysis of anti-dyn (*n* = 8 cells) or GST-Dyn_829-842_ (*n* = 5 cells). (iii) Replenishment of ETAP control conditions with 1.8 mM Ca^2+^/70 μM EGTA (black filled circles), when an anti-dynamin antibody was added to the internal solution (red filled circles), and in presence of GST-Dyn_829-842_ (green open circles). The averaged control values were fitted (dotted line) to a monoexponential growing function (R = R_0_ + A.(1 - e^
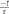
^)), obtaining a value at time zero R_0_ of 0.15 ± 0.07, an asymptote A of 0.87 ± 0.07, a time constant τ of 1.04 ± 0.20 s, and a correlation coefficient *R* > 0.9867. ^∗^*p* < 0.05; ^∗∗^*p* < 0.001; ^∗∗∗^*p* < 0.0002.

We also studied the replenishment of ETAP in 1.8 mM external Ca^2+^/70 μM internal EGTA, which better represent the physiological extracellular Ca^2+^ concentration and intracellular Ca^2+^ buffering conditions, respectively (Neher and Augustine, 1992). As indicated previously (**Figures 3** and **4**), in this experimental condition I_Ca2+_ and ΔC_m_ were smaller (247 ± 21 pA; 8 ± 1 fF; *n* = 21) than in the previous standard situation with 5 mM external Ca^2+^/0.5 mM internal EGTA. However, the fast endocytotic process still showed similar kinetics than our previous condition, and compensated completely the previous exocytosis (**Figures 9Ai,ii**). Additionally, ETAP was replenished rapidly (**Figure 9Biii**, black filled circles), with kinetics comparable with the one obtained at higher Ca^2+^ and EGTA concentrations. The effect of inhibition of dynamin was also tested. Since anti-Dyn and GST-Dyn reduce I_Ca2+_ in approximately 30% (see above), we dialyzed our cells with an internal solution containing 70 μM EGTA and either anti-Dyn or GST-Dyn, in presence of 5 mM external Ca^2+^. We decided to do the experiments in these conditions with the aim to reach similar I_Ca2+_ in the three experimental groups (control, anti-Dyn, and GST-Dyn). Indeed, we obtained I_Ca2+_ values of 269 ± 43 and 248 ± 24 pA for anti-Dyn and GST-Dyn, respectively; such values were comparable to the control condition (1.8 mM external Ca^2+^ and 70 μM internal EGTA), whereas in cells bathed in 1.8 or 3 mM external Ca^2+^, the application of anti-Dyn reduced I_Ca2+_ to 143,2 ± 17.3 pA (*n* = 5) or 206.6 ± 43.2 pA (*n* = 5), respectively. The exocytosis in presence of 5 mM external Ca^2+^ was also comparable with the control condition, with ΔC_m_ values of 7.1 ± 0.3 and 7.7 ± 1.8 fF (*n* = 11), for anti-Dyn and GST-Dyn, respectively. Importantly, the application of anti-Dyn or GST-Dyn inhibited significantly the endocytosis and delayed ETAP replenishment (**Figures 9Bi–iii**, red filled circles and green opened circles, respectively). These experiments reaffirm our previous conclusions about the presence of a rapid replenishment mechanism in chromaffin cells and its dependence on a dynamin dependent endocytosis.

### Synchronous Exocytosis during Repetitive AP_ls_ Stimulation

**FIGURE 10:**
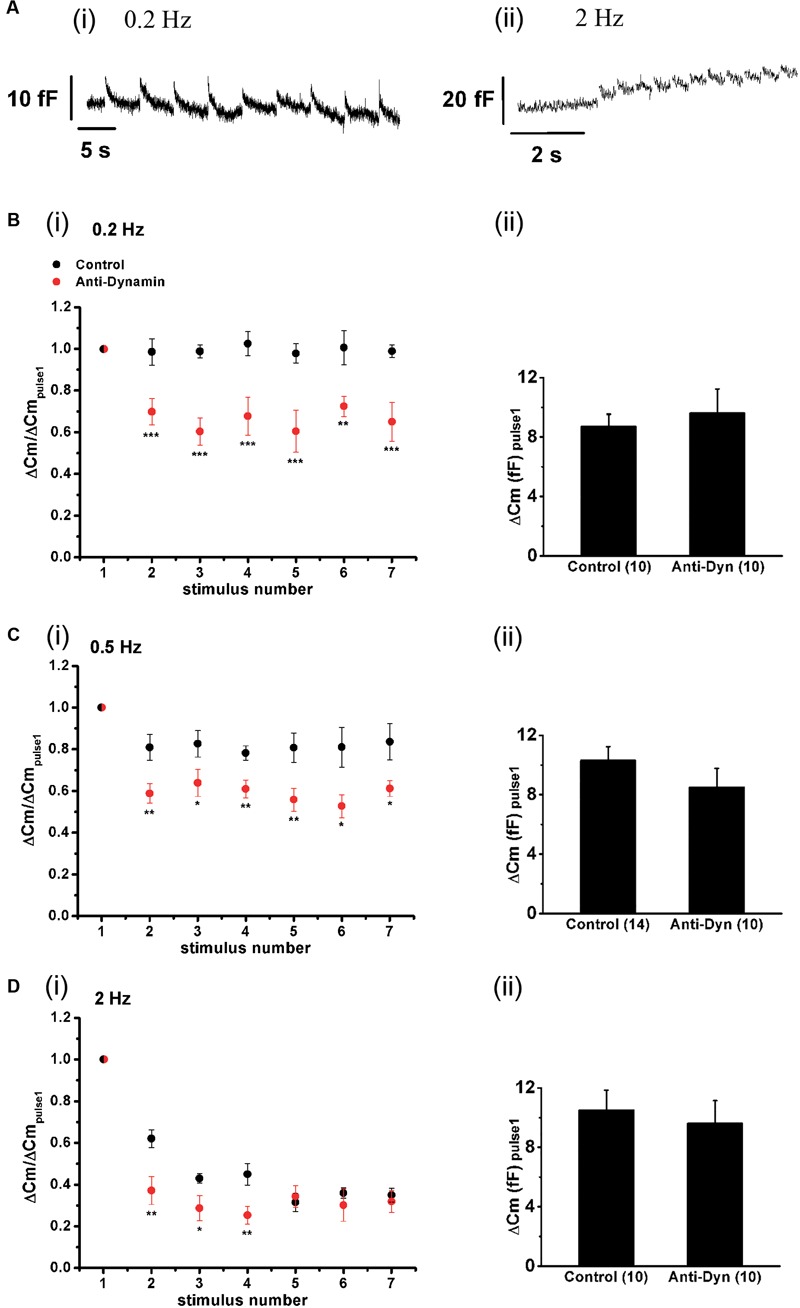
**Effect of the stimulation rate and the inhibition of dynamin on synchronous exocytosis during short AP_ls_ trains. (A)** Original recordings of capacitance changes during trains of AP_ls_ at 0.2 (i) and 2 Hz (ii). **(B–D)** (i) Synchronous capacitance changes obtained between the 1^st^ and the 7^th^ AP_ls_ of trains applied in control conditions (black circles) and in presence of the anti-dynamin antibody (red circles) for 0.2, 0.5, and 2 Hz, respectively (^∗^*p* < 0.05; ^∗∗^*p* < 0.02; and ^∗∗∗^*p* < 0.005 represent the statistical significance between control and Anti-Dyn for each stimulus number). The measurements obtained in each individual experiment were normalized respect to the exocytosis induced by the 1^st^ AP_ls_ of the train. (ii) The bar diagrams represent the absolute values of the synchronous exocytosis induced by the 1^st^ AP_ls_ of the trains, in control condition and in presence of the antibody. The number of experiments in each condition is represented between parentheses at the base of the bar diagrams.

The replenishment rate of a vesicular pool is critical for its exocytotic performance during maintained stimulation (Voets et al., 1999; Van Hook et al., 2014). The experiments of the previous sections indicate that ETAP is replenished in a very short period. From the estimated time constant (τ = 0.73 s), we can predict that the exocytosis induced by each AP_ls_ will be kept approximately constant along the train if chromaffin cells are stimulated with frequencies lower than 0.5 Hz (interval between stimuli >3 τ). To evaluate this prediction, we applied trains of 10 AP_ls_ at 0.2, 0.5, and 2 Hz, and analyzed the synchronous exocytosis, defined as the difference between the mean C_m_ measured in a 100 ms window starting 60 ms after the end of each AP_ls_ minus the mean pre-stimulus capacitance also measured in a 100 ms window. Data represented in **Figure 10** clearly show that synchronous exocytosis did not decrease noticeably during the application of a short train of AP_ls_ at 0.2 Hz (**Figure 10Ai**, and black circles in **Figure 10Bi**). Synchronous exocytosis during 0.5 Hz trains showed a small (approximately 20%) decrease (*p* < 0.05) in the second AP_ls_, and then remained stable for the rest of the train (ANOVA between 2, 3, 4, 5, 6, and 7 stimuli was not significant, *p* > 0.85; black circles in **Figure 10Ci**). In contrast, the application of trains at 2 Hz provoked more than 50% decrease in synchronous exocytosis (*p* < 0.0001) (black circles, **Figure 10Di**), These results are in agreement with the kinetics of ETAP replenishment described above, suggesting that this replenishment process can maintain by itself synchronous exocytosis at low frequencies.

To investigate the effect of blocking the fast endocytosis on the maintenance of synchronous exocytosis during brief train stimulation, we introduced anti-Dyn in the internal solution, wait 5 min for diffusion, and started with train stimulation protocols. It is important to mention that the application of the antibody did not affect the absolute values of exocytosis recorded during the first AP_ls_ of the trains at every frequency (bar diagrams in **Figures 10Bii,Cii,Dii**). In contrast, the considerable inhibition of the rapid ETAP replenishment induced by this treatment (**Figure 8Biii**, red opened circles) interfered in the exocytotic performance of chromaffin cells during train stimulation. The red circles in **Figures 10Bi,Ci,Di** represent the synchronous exocytosis associated to these experiments. The results show that anti-Dyn provoked a significant decrease of synchronous exocytosis at 0.2, 0.5, and 2 Hz stimulation frequencies in comparison with control experiments (see **Figure 10** legend for statistics).

**FIGURE 11:**
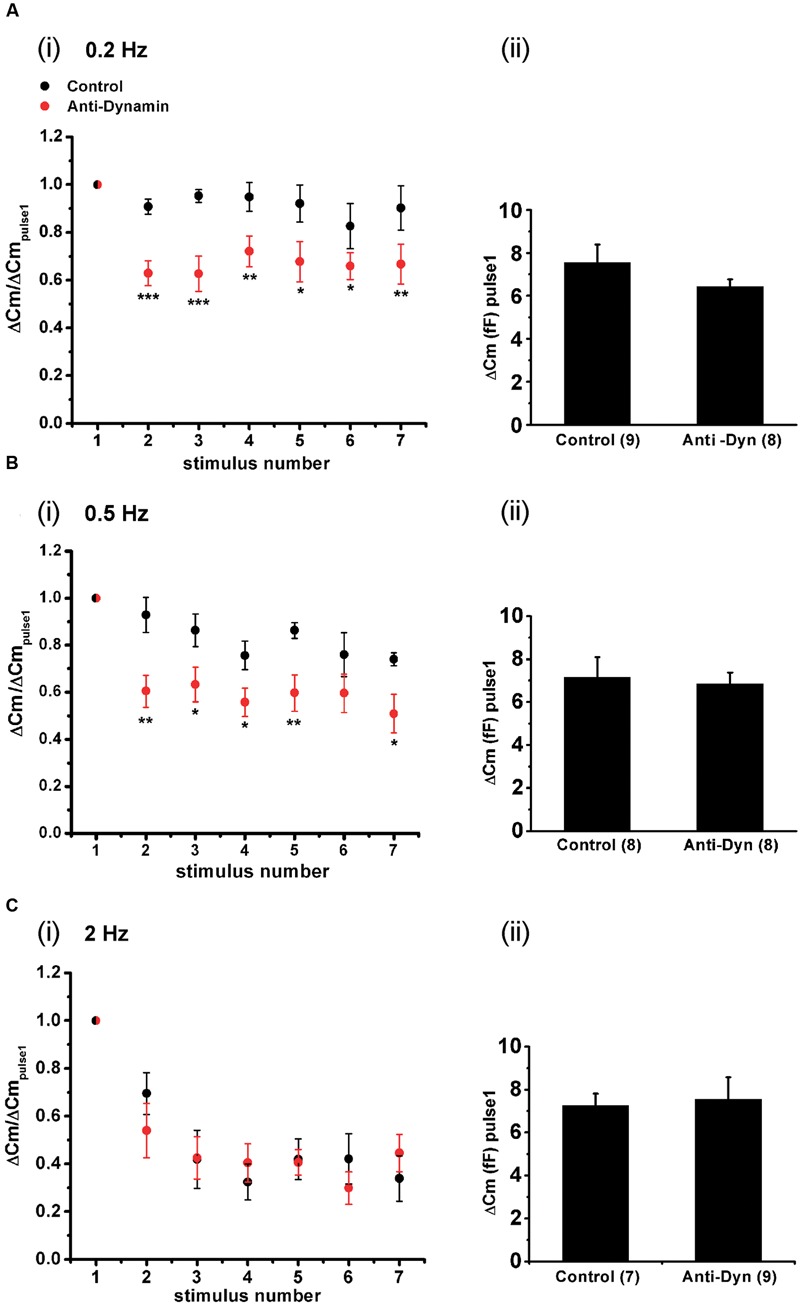
**Effect of the stimulation rate during short AP_ls_ trains applied at physiological Ca^2+^. (A–C)** (i) Synchronous capacitance changes obtained between the 1^st^ and the 7^th^ AP_ls_ of trains applied in control conditions using 1.8 mM external Ca^2+^/70 μM internal EGTA (black circles), and in presence of the anti-dynamin antibody (red circles) for 0.2, 0.5, and 2 Hz, respectively (^∗^*p* < 0.05; ^∗∗^*p* < 0.01; and ^∗∗∗^*p* < 0.001 represent the statistical significance between control and Anti-Dyn for each stimulus number). The measurements obtained in each individual experiment were normalized respect to the exocytosis induced by the 1^st^ AP_ls_ of the train. (ii) The bar diagrams represent the absolute values of the synchronous exocytosis induced by the 1^st^ AP_ls_ of the trains, in control condition and in presence of the antibody. The number of experiments in each condition is represented between parentheses at the base of the bar diagrams.

Finally, we also analyzed the maintenance of synchronous exocytosis during APls train stimulation in presence of 1.8 mM external Ca^2+^/70 μM internal EGTA. Synchronous exocytosis was maintained stable during the application of AP_ls_ train stimulation at 0.2 Hz, and suffered a moderate decrease at 0.5 Hz. Conversely, at 2 Hz, we observed a notorious decrease in synchronous exocytosis to approximately 35% of initial value. The application of anti-Dyn affected significantly the maintenance of synchronous exocytosis at 0.2 and 0.5 Hz stimulation frequencies (**Figure 11**).

## Discussion

Immediately releasable pool is a pool of vesicles highly coupled to VDCC (Voets et al., 1999; Alvarez et al., 2013), which is selectively released by the application of short depolarizations (Horrigan and Bookman, 1994). The relative contribution of this pool of vesicles to the exocytosis at high stimulation frequencies or prolonged depolarizations, which mainly promote the exocytosis of vesicles poorly coupled to VDCC, is negligible (Chow et al., 1992; Zhou and Misler, 1995; Voets et al., 1999). Conversely, IRP importantly contributes to the exocytosis induced by isolated action potentials (**Figure 2B**). Nevertheless, an efficient vesicle replenishment mechanism is required to maintain the catecholamine release during repeated action potentials, even at low frequency, like in basal sympathetic tone. In the present work, we analyzed the rate of replenishment of immediately releasable vesicles in different conditions of stimulation. We found that when IRP was depleted completely by a 50 ms depolarization, the refilling rate of this pool was slow and non-compatible with physiological firing frequencies. However, stimulation of chromaffin cells with single AP_ls_ allows rapid replenishment of vesicles (**Figures 5** and **9**), consistent with the maintenance of synchronous exocytosis during applications of short trains of AP_ls_ at low frequencies (0.2 or 0.5 Hz) (**Figures 10** and **11**). We identified two processes participating in such rapid vesicle replenishment: (i) the transfer of vesicles from RRP and (ii) a dynamin-dependent fast endocytosis.

It is important to note that the degree of coupling between vesicles and channels, and therefore synchronicity of exocytosis with stimulus, in chromaffin cells is lower than in neurons, even for IRP. While neurons can present a latency of release lower than 2 ms, chromaffin cells stimulated at low action potential frequencies presents a latency of more than 10 ms (Zhou and Misler, 1995).

The refilling of IRP was successfully fitted to a single exponential growing function with a time constant of 7.5 s (**Figure 1**). This refilling rate is consistent with previous reports of IRP replenishment in mouse adrenal slices (Moser and Neher, 1997b) and of RRP refilling in chromaffin cells and synaptic terminals (von Gersdorff and Matthews, 1997; Smith et al., 1998; Voets et al., 1999). This time constant indicates that the replenishment of IRP cannot be completed in less than 20 s, and therefore firing frequencies higher than 0.05 Hz will provoke the depletion of this pool. However, it must be considered that several factors have been reported to affect RRP or IRP replenishment, for example temperature (Dinkelacker et al., 2000), cytosolic Ca^2+^ concentration (Smith et al., 1998), PKC activity (Gillis et al., 1996), Doc2b (Pinheiro et al., 2013), CAPS (Liu et al., 2008), and NCAM (Chan et al., 2005a), and probably some of them may contribute to tune the IRP replenishment according to physiological requirements.

According to our data, ETAP (approximately 11 fF) is a part of what we define as IRP (see **Figure 4**). Since the average capacitance of one vesicle was estimated to be 1.3 fF (Moser and Neher, 1997a), ETAP would be equivalent to approximately eight vesicles, corresponding roughly to a 40% of the total IRP. However, when we studied the replenishment of ETAP, our results indicated that this group of vesicles does not behave like total IRP. ETAP was replenished very rapidly (τ 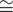 0.75 s), approximately one order faster than total IRP refilling (**Figures 5** and **9**). In summary, ETAP would represent a small and fast component of release, which can be replenished quickly, as it was proposed in some synapse preparations (Cho et al., 2011).

The replenishment kinetics of ETAP is fast enough to replenish completely ETAP during the intervals between AP_ls_ applied at low frequencies, which are in the range of frequencies of native action potentials in basal physiological conditions (Kidokoro and Ritchie, 1980; Chan and Smith, 2001). Moreover, this replenishment kinetics is consistent with the behavior of synchronous exocytosis during trains of AP_ls_ at different frequencies (**Figures 10** and **11**). Frequencies below 0.5 Hz, which have an inter-stimulus period larger than three time constants, did not provoke a significant decrease in synchronous exocytosis. On the other hand, the application of AP_ls_ at 2 Hz decreased synchronous exocytosis to approximately 40% of the initial values. These results indicate that ETAP by itself can maintain exocytosis during low stimulation frequencies. In a recent paper, using amperometry, Lefkowitz et al. (2014) proposed a mechanism by which spontaneous asynchronous exocytosis may contribute importantly to secretion during action potential waveforms applied at 0.5 Hz. We did not observe evident signs of asynchronous exocytosis during several seconds after the application of AP_ls_ (see **Figure 8**). The noise in our measurements would not allow the detection of single vesicle fusion, but important asynchronous exocytosis, as the one detected by Lefkowitz et al. (2014), should interfere in the monotonic decrease in capacitance, which is characteristic of our measurements. However, we cannot discard that asynchronous exocytosis may have a more significant contribution during prolonged trains of action potentials at low frequencies. Furthermore, it is possible that both mechanisms contribute to the maintenance of secretion during basal firing frequencies in chromaffin cells.

**FIGURE 12:**
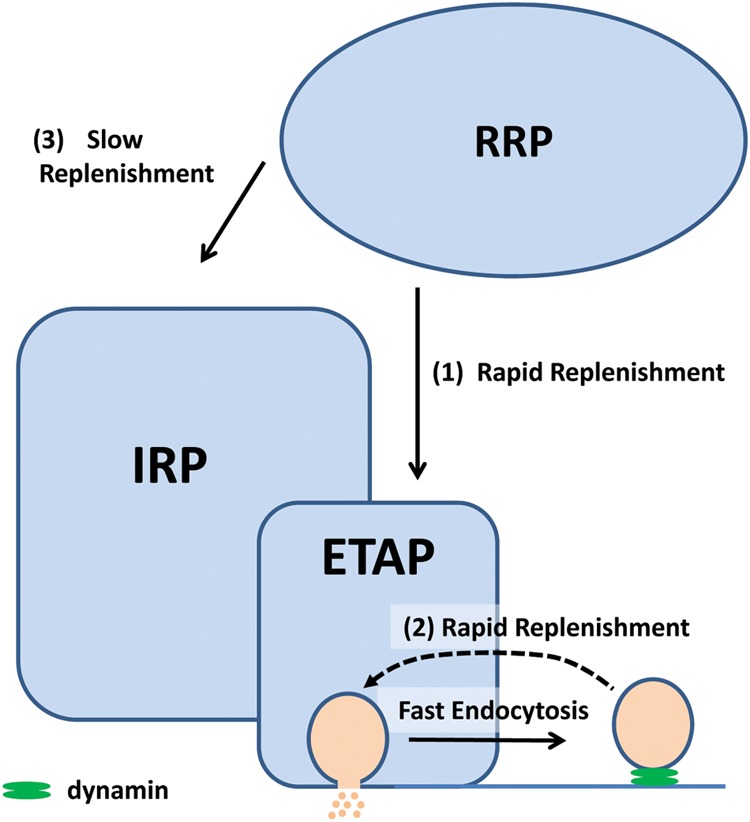
**We proposed that in chromaffin cells, the exocytosis of a subpopulation of vesicles of the IRP is triggered by single action potential (ETAP).** This group of vesicles is replenished rapidly via two mechanisms: (1) by transferring vesicles from the RRP and (2) by a dynamin-dependent fast endocytosis. Fast endocytosis could allow the direct vesicle recovery via a kiss-and-run like mechanism (Fulop et al., 2005), or alternatively, it might restore the structure of the release sites (Hosoi et al., 2009). These mechanisms would explain the sustained exocytosis at physiological basal action potential frequency in these cells. The RRP can also supply vesicles, with a slow kinetics (3), to the rest of IRP. The relative sizes in this cartoon do not have relationship with the real magnitude of the pools.

We considered three alternative hypotheses that might explain the fast ETAP replenishment. The first one assumed that ETAP was refilled directly from IRP. The second one, that ETAP was replenished by transfer of vesicles from RRP. Finally, a third possibility considered that a fast endocytotic process, directly coupled to exocytosis, is the first step of vesicle replenishment. The results obtained in this work show that when IRP was severely depleted by a 50 ms depolarization pulse, ETAP was still replenished very rapidly, rejecting the first hypothesis. In contrast, the depletion of RRP significantly delayed ETAP replenishment (**Figure 7**). Therefore, the fast replenishment of ETAP can be at least partially explained by transfer of vesicles from RRP [pathway (1) in **Figure 12**]. In a previous work, Chan et al. (2005a) demonstrated in mouse adrenal medulla slices that IRP vesicles can be refilled rapidly from RRP. Since ETAP is a subpopulation of IRP, it is probable that in our preparation this fast mechanism of refilling operates only on this subpopulation. It is well known from previous classical papers that the size of RRP depends on the basal cytosolic Ca^2+^ concentration, reaching a maximum size between 400 and 600 nM Ca^2+^, and decreasing at higher Ca^2+^ concentrations, probably because of spontaneous exocytosis (von Ruden and Neher, 1993; Voets, 2000). In agreement with this behavior of RRP, the time constant of ETAP replenishment showed a tendency to increase at 600 nM and to decrease at 900 nM Ca^2+^. Therefore, it is possible that the steady size of RRP might affect the speed of ETAP replenishment.

ETAP replenishment after RRP depletion is still faster than total IRP refilling (compare open circles with the dashed line in **Figure 7B**), making possible the participation of an additional mechanism in fast vesicle replenishment. We observed that ETAP was consistently followed by a fast endocytosis that completely compensated the preceding exocytosis (**Figures 8** and **9**). This endocytotic process has a kinetics similar to that described by Smith et al. (1998) in chromaffin cells (Chan and Smith, 2001). In contrast, the application of a 50 ms pulse, which promotes total IRP exocytosis, did not result in a fast endocytotic process, being consistent with the results of Engisch and Nowycky (1998). We hypothesized that the fast endocytosis developed after ETAP contributes to the rapid vesicle replenishment process. To address this possibility, we applied two treatments that resulted in a strong inhibition of fast endocytosis: (1) the cellular dialysis of a monoclonal antibody against dynamin (Artalejo et al., 2002), and (2) the intracellular application of the peptide GST-Dyn_829-842_ that disrupts dynamin-dependent endocytosis (Shupliakov et al., 1997; Fulop et al., 2008). Remarkably, these treatments severely delayed the ETAP replenishment (**Figures 8** and **9**). These results lead us to conclude that the replenishment of ETAP is tightly coupled to a dynamin-dependent fast endocytotic process [pathway (2) in **Figure 12**]. Various authors have proposed the existence of local vesicle recycling mechanisms, in where the vesicles are recovered after partial fusion (Cardenas and Marengo, 2010). The presence of kiss-and–run and cavicapture exocytosis mechanisms in chromaffin cells has been postulated by different authors (Ales et al., 1999; Taraska et al., 2003; Perrais et al., 2004; Elhamdani et al., 2006; Gonzalez-Jamett et al., 2010), and it was proposed that kiss-and-run correlates with a dynamin-dependent rapid endocytosis (Elhamdani et al., 2006; Wu et al., 2014). Fulop et al. (2005) and Fulop and Smith (2006) postulated that basal firing rates of action potentials result in an Ω-shape kiss-and-run like fusion event, while high frequencies promotes full granule collapse. Therefore, it is possible that the application of AP_ls_ individually or at low frequencies promotes a kiss-and-run like process, which results in rapid ETAP replenishment. On the other hand, inhibition of dynamin would interrupt the fission of partially fused vesicles, blocking fast endocytosis and delaying ETAP replenishment. Although the kiss-and–run mechanism is an attractive hypothesis, from our results, we cannot rule out the possibility that rapid endocytosis recovers membrane to an intermediate, non-releasable compartment, from where mature releasable vesicles are produced. In this direction, an alternative hypothesis was proposed by the groups of Takeshi Sakaba and Ling-Gang Wu in the calyx of Held (Hosoi et al., 2009; Wu and Wu, 2009; Wu et al., 2014). According with that hypothesis, after exocytosis, a fast endocytotic mechanism may facilitate vesicle replenishment by clearance of exocytotic materials from active zones, restoring the structure of the release sites. Simultaneously, the vesicles retrieved by fast endocytosis would recycle to a large recycling pool to prevent vesicle exhaustion, and from where mature releasable vesicles can be produced.

If the rapid replenishment of ETAP is coupled with fast endocytosis, the inhibition of vesicle fission process, by blocking dynamin, should affect the maintenance of synchronous exocytosis during trains of AP_ls_ applied at low frequencies. In agreement with this assumption, the application of the anti-dynamin antibody provoked a significant decrease in synchronous exocytosis during repetitive AP_ls_ stimulation at low frequencies (0.2 and 0.5 Hz, in **Figures 10B,C**, respectively, and **Figures 11A,B**, respectively).

In summary, we found that both the transfer of vesicles from RRP and fast endocytosis contribute to the replenishment of ETAP. It is possible that these two mechanisms represent steps of two independent pathways of vesicle replenishment. In agreement with this hypothesis, there was an additive effect on the inhibition of ETAP replenishment when these two processes were blocked together (**Figure 8Biii**, green diamonds). The residual refilling obtained in this condition might be explained by a not complete depletion of RRP with the double 100 ms depolarization protocol, or a slower replenishment process from upstream pools. However, we cannot rule out the possibility that fast endocytosis and transfer of vesicles from RRP are both single steps of the same path. Future investigations will be necessary to discriminate between these two alternatives.

In stress conditions chromaffin cells fire action potentials at high frequencies, promoting accumulation of cytosolic residual Ca^2+^ and massive exocytosis irrespective of the location of secretory vesicles respect to VDCC (Duan et al., 2003). Such high frequencies favors asynchronous over synchronous exocytosis (Zhou and Misler, 1995). On the opposite side, in rest conditions the firing frequency is low, Ca^2+^ does not accumulate between stimuli, and exocytosis would be limited to vesicles closely coupled to VDCC (Voets et al., 1999; Alvarez and Marengo, 2011). So, it is likely that the physiological importance of IRP resides in its highly efficient stimulus-exocytosis coupling, which allows secretion during action potentials at low rate (Oré and Artalejo, 2005; Cardenas and Marengo, 2016). If this is the case, IRP would need a rapid replenishment mechanism compatible with physiological basal action potential frequencies. The rapid replenishment of ETAP might be the solution of this problem. According to our results, secretory vesicles are replenished rapidly after ETAP depletion (τ < 1 s), increasing the probability of release of the cell in response to a new AP_ls_. In our experimental conditions the replenishment of ETAP is fast enough to account for an AP_ls_ frequency of 0.2–0.5 Hz, which is approximately the physiological basal frequency in chromaffin cells. However, in chromaffin cells *in situ*, physiological variables, such as hormones, cytokines, and other released transmitters, might modify this process. The understanding of the regulatory mechanisms and variables that control this fast vesicle replenishment process should be the subject of next investigations in the field.

## Author Contributions

JM-D: conducted animal surgeries, designed and performed experiments, interpreted results, performed statistical analysis and critically revised the manuscript. YÁ: conducted animal surgeries, designed and performed experiments, interpreted results and performed statistical analysis. MM, LB, and AB: conducted animal surgeries, performed experiments, interpreted results and performed statistical analysis. AG-J: conducted animal surgeries, designed and performed experiments, interpreted results, performed statistical analysis and critically revised the manuscript. AC: designed experiments, interpreted results, helped draft parts of the manuscript and critically revised the manuscript. FM: designed experiments, interpreted results, performed statistical analysis, conceived the study, and draft the manuscript. All authors red and approved the final manuscript.

## Conflict of Interest Statement

The authors declare that the research was conducted in the absence of any commercial or financial relationships that could be construed as a potential conflict of interest.

## Abbreviations

AntiDyn: anti-dynamin monoclonal antibody
APls: action potential like stimulus
C_m_: membrane capacitance
ETAP: exocytosis triggered by action potential like stimulus
I_Ca2+_: calcium currents
IRP: immediately releasable pool
RRP: ready releasable pool
VDCC: voltage dependent calcium channels

## References

[B1] Ales E. Tabares L. Poyato J. M. Valero V. Lindau M. Alvarez De Toledo T. (1999). High calcium concentrations shift the mode of exocytosis to the kiss-and-run mechanism. *Nat. Cell Biol.* 1 40–44. 10559862 10.1038/9012

[B2] Alvarez Y. D. Belingheri V. A. Perez Bay A. E. Javis S. E. Tedford H. W. Zamponi G. (2013). The immediately releasable pool of mouse chromaffin cell vesicles is coupled to p/q-type calcium channels via the synaptic protein interaction site. *PLoS ONE* 8:e54846 10.1371/journal.pone.0054846 PMC3559834 23382986

[B3] Alvarez Y. D. Ibanez L. I. Uchitel O. D. Marengo F. D. (2008). P/Q Ca2+ channels are functionally coupled to exocytosis of the immediately releasable pool in mouse chromaffin cells. *Cell Calcium* 43 155–164. 10.1016/j.ceca.2007.04.014 17561253

[B4] Alvarez Y. D. Marengo F. D. (2011). The immediately releasable vesicle pool: highly coupled secretion in chromaffin and other neuroendocrine cells. *J. Neurochem.* 116 155–163. 10.1111/j.1471-4159.2010.07108.x 21073467

[B5] Artalejo C. R. Elhamdani A. Palfrey H. C. (2002). Sustained stimulation shifts the mechanism of endocytosis from dynamin-1-dependent rapid endocytosis to clathrin- and dynamin-2-mediated slow endocytosis in chromaffin cells. *Proc. Natl. Acad. Sci. U.S.A.* 99 6358–6363. 10.1073/pnas.082658499 11959911 PMC122953

[B6] Brandt B. L. Hagiwara S. Kikodoro Y. Miyazaki S. (1976). Action potentials in the rat chromaffin cell and effects of acetylcholine. *J. Physiol.* 263 417–439. 10.1113/jphysiol.1976.sp011638 1018274 PMC1307710

[B7] Burgoyne R. D. (1998). Two forms of triggered endocytosis in regulated secretory cells. *J. Physiol.* 506(Pt 3) 589 10.1111/j.1469-7793.1998.589bv.x PMC2230748 9503323

[B8] Cardenas A. M. Marengo F. D. (2010). Rapid endocytosis and vesicle recycling in neuroendocrine cells. *Cell. Mol. Neurobiol.* 30 1365–1370. 10.1007/s10571-010-9579-8 21046457 PMC11498848

[B9] Cardenas A. M. Marengo F. D. (2016). How the stimulus defines the dynamics of vesicle pool recruitment, fusion mode and vesicle recycling in neuroendocrine cells. *J. Neurochem.* 137 867–879. 10.1111/jnc.13565 26849771

[B10] Chan S. A. Polo-Parada L. Landmesser L. T. Smith C. (2005a). Adrenal chromaffin cells exhibit impaired granule trafficking in NCAM knockout mice. *J. Neurophysiol.* 94 1037–1047. 10.1152/jn.01213.2004 15800072

[B11] Chan S. A. Polo-Parada L. Smith C. (2005b). Action potential stimulation reveals an increased role for P/Q-calcium channel-dependent exocytosis in mouse adrenal tissue slices. *Arch. Biochem. Biophys.* 435 65–73. 10.1016/j.abb.2004.12.005 15680908

[B12] Chan S. A. Smith C. (2001). Physiological stimuli evoke two forms of endocytosis in bovine chromaffin cells. *J. Physiol.* 537 871–885. 10.1113/jphysiol.2001.012838 11744761 PMC2279013

[B13] Chan S. A. Smith C. (2003). Low frequency stimulation of mouse adrenal slices reveals a clathrin-independent, protein kinase C-mediated endocytic mechanism. *J. Physiol.* 553 707–717. 10.1113/jphysiol.2003.053918 14500763 PMC2343636

[B14] Cho S. Li G. L. von Gersdorff H. (2011). Recovery from short-term depression and facilitation is ultrafast and Ca2+ dependent at auditory hair cell synapses. *J. Neurosci.* 31 5682–5692. 10.1523/JNEUROSCI.5453-10.2011 21490209 PMC3090423

[B15] Chow R. H. Klingauf J. Heinemann C. Zucker R. S. Neher E. (1996). Mechanisms determining the time course of secretion in neuroendocrine cells. *Neuron* 16 369–376. 10.1016/S0896-6273(00)80054-9 8789951

[B16] Chow R. H. von Ruden L. Neher E. (1992). Delay in vesicle fusion revealed by electrochemical monitoring of single secretory events in adrenal chromaffin cells. *Nature* 356 60–63. 10.1038/356060a0 1538782

[B17] Dinkelacker V. Voets T. Neher E. Moser T. (2000). The readily releasable pool of vesicles in chromaffin cells is replenished in a temperature-dependent manner and transiently overfills at 37 degrees C. *J. Neurosci.* 20 8377–8383. 11069944 10.1523/JNEUROSCI.20-22-08377.2000 PMC6773192

[B18] Duan K. Yu X. Zhang C. Zhou Z. (2003). Control of secretion by temporal patterns of action potentials in adrenal chromaffin cells. *J. Neurosci.* 23 11235–11243. 14657183 10.1523/JNEUROSCI.23-35-11235.2003 PMC6741046

[B19] Elhamdani A. Azizi F. Artalejo C. R. (2006). Double patch clamp reveals that transient fusion (kiss-and-run) is a major mechanism of secretion in calf adrenal chromaffin cells: high calcium shifts the mechanism from kiss-and-run to complete fusion. *J. Neurosci.* 26 3030–3036. 10.1523/JNEUROSCI.5275-05.2006 16540581 PMC6673983

[B20] Engisch K. L. Nowycky M. C. (1998). Compensatory and excess retrieval: two types of endocytosis following single step depolarizations in bovine adrenal chromaffin cells. *J. Physiol.* 506 591–608. 10.1111/j.1469-7793.1998.591bv.x 9503324 PMC2230744

[B21] Fulop T. Doreian B. Smith C. (2008). Dynamin I plays dual roles in the activity-dependent shift in exocytic mode in mouse adrenal chromaffin cells. *Arch. Biochem. Biophys.* 477 146–154. 10.1016/j.abb.2008.04.039 18492483 PMC2593866

[B22] Fulop T. Radabaugh S. Smith C. (2005). Activity-dependent differential transmitter release in mouse adrenal chromaffin cells. *J. Neurosci.* 25 7324–7332. 10.1523/JNEUROSCI.2042-05.2005 16093382 PMC6725304

[B23] Fulop T. Smith C. (2006). Physiological stimulation regulates the exocytic mode through calcium activation of protein kinase C in mouse chromaffin cells. *Biochem. J.* 399 111–119. 10.1042/BJ20060654 16784416 PMC1570168

[B24] Gillis K. D. (1995). “Techniques for membrane capacitance measurements,” in *Single-Channel Recording* 2nd Edn eds Sakmann B. Neher E. (New York, NY: Plenum Press) 155–198. 10.1007/978-1-4419-1229-9_7

[B25] Gillis K. D. Mossner R. Neher E. (1996). Protein kinase C enhances exocytosis from chromaffin cells by increasing the size of the readily releasable pool of secretory granules. *Neuron* 16 1209–1220. 10.1016/S0896-6273(00)80147-6 8663997

[B26] Gong L. W. Di Paolo G. Diaz E. Cestra G. Diaz M. E. Lindau M. (2005). Phosphatidylinositol phosphate kinase type I gamma regulates dynamics of large dense-core vesicle fusion. *Proc. Natl. Acad. Sci. U.S.A.* 102 5204–5209. 10.1073/pnas.0501412102 15793002 PMC555604

[B27] Gonzalez-Gutierrez G. Miranda-Laferte E. Neely A. Hidalgo P. (2007). The Src homology 3 domain of the beta-subunit of voltage-gated calcium channels promotes endocytosis via dynamin interaction. *J. Biol. Chem.* 282 2156–2162. 10.1074/jbc.M609071200 17110381

[B28] Gonzalez-Jamett A. M. Baez-Matus X. Hevia M. A. Guerra M. J. Olivares M. J. Martinez A. D. (2010). The association of dynamin with synaptophysin regulates quantal size and duration of exocytotic events in chromaffin cells. *J. Neurosci.* 30 10683–10691. 10.1523/JNEUROSCI.5210-09.2010 20702699 PMC6634707

[B29] Grabs D. Slepnev V. I. Songyang Z. David C. Lynch M. Cantley L. C. (1997). The SH3 domain of amphiphysin binds the proline-rich domain of dynamin at a single site that defines a new SH3 binding consensus sequence. *J. Biol. Chem.* 272 13419–13425. 10.1074/jbc.272.20.13419 9148966

[B30] Harata N. C. Aravanis A. M. Tsien R. W. (2006). Kiss-and-run and full-collapse fusion as modes of exo-endocytosis in neurosecretion. *J. Neurochem.* 97 1546–1570. 10.1111/j.1471-4159.2006.03987.x 16805768

[B31] Holman M. E. Coleman H. A. Tonta M. A. Parkington H. C. (1994). Synaptic transmission from splanchnic nerves to the adrenal medulla of guinea-pigs. *J. Physiol.* 478 115–124. 10.1113/jphysiol.1994.sp020235 7965827 PMC1155650

[B32] Horrigan F. T. Bookman R. J. (1994). Releasable pools and the kinetics of exocytosis in adrenal chromaffin cells. *Neuron* 13 1119–1129. 10.1016/0896-6273(94)90050-7 7946349

[B33] Hosoi N. Holt M. Sakaba T. (2009). Calcium dependence of exo- and endocytotic coupling at a glutamatergic synapse. *Neuron* 63 216–229. 10.1016/j.neuron.2009.06.010 19640480

[B34] Kidokoro Y. Ritchie A. K. (1980). Chromaffin cell action potentials and their possible role in adrenaline secretion from rat adrenal medulla. *J. Physiol.* 307 199–216. 10.1113/jphysiol.1980.sp013431 7205664 PMC1283041

[B35] Lefkowitz J. J. DeCrescenzo V. Duan K. Bellve K. D. Fogarty K. E. Walsh J. V. (2014). Catecholamine exocytosis during low frequency stimulation in mouse adrenal chromaffin cells is primarily asynchronous and controlled by the novel mechanism of Ca2+ syntilla suppression. *J. Physiol.* 592 4639–4655. 10.1113/jphysiol.2014.278127 25128575 PMC4253468

[B36] Liu Y. Schirra C. Stevens D. R. Matti U. Speidel D. Hof D. (2008). CAPS facilitates filling of the rapidily releasable pool of large dense-core vesicles. *J. Neurosci.* 28 5594–5601. 10.1523/JNEUROSCI.5672-07.2008 18495893 PMC6670619

[B37] Marengo F. D. (2005). Calcium gradients and exocytosis in bovine adrenal chromaffin cells. *Cell Calcium* 38 87–99. 10.1016/j.ceca.2005.06.006 16076487

[B38] Marengo F. D. Monck J. R. (2003). Spatial distribution of Ca(2+) signals during repetitive depolarizing stimuli in adrenal chromaffin cells. *Biophys. J.* 85 3397–3417. 10.1016/S0006-3495(03)74759-6 14581241 PMC1303617

[B39] Moser T. Neher E. (1997a). Estimation of mean exocytic vesicle capacitance in mouse adrenal chromaffin cells. *Proc. Natl. Acad. Sci. U.S.A.* 94 6735–6740. 10.1073/pnas.94.13.6735 9192634 PMC21227

[B40] Moser T. Neher E. (1997b). Rapid exocytosis in single chromaffin cells recorded from mouse adrenal slices. *J. Neurosci.* 17 2314–2323. 9065492 10.1523/JNEUROSCI.17-07-02314.1997 PMC6573505

[B41] Neher E. (1998). Vesicle pools and Ca2+ microdomains: new tools for understanding their roles in neurotransmitter release. *Neuron* 20 389–399. 10.1016/S0896-6273(00)80983-6 9539117

[B42] Neher E. Augustine G. J. (1992). Calcium gradients and buffers in bovine chromaffin cells. *J. Physiol.* 450 273–301. 10.1113/jphysiol.1992.sp019127 1331424 PMC1176122

[B43] Oré L. O. Artalejo A. R. (2005). Intracellular Ca2+ microdomain-triggered exocytosis in neuroendocrine cells. *Trends Neurosci.* 27 113–115. 10.1016/j.tins.2004.01.001 15046078

[B44] Perez Bay A. E. Belingheri A. V. Alvarez Y. D. Marengo F. D. (2012). Membrane cycling after the excess retrieval mode of rapid endocytosis in mouse chromaffin cells. *Acta Physiol. (Oxf.)* 204 403–418. 10.1111/j.1748-1716.2011.02340.x 21791014

[B45] Perrais D. Kleppe I. C. Taraska J. W. Almers W. (2004). Recapture after exocytosis causes differential retention of protein in granules of bovine chromaffin cells. *J. Physiol.* 560 413–428. 10.1113/jphysiol.2004.064410 15297569 PMC1665250

[B46] Pinheiro P. S. de Wit H. Walter A. M. Groffen A. J. Verhage M. Sorensen J. B. (2013). Doc2b synchronizes secretion from chromaffin cells by stimulating fast and inhibiting sustained release. *J. Neurosci.* 33 16459–16470. 10.1523/JNEUROSCI.2656-13.2013 24133251 PMC6618527

[B47] Pusch M. Neher E. (1988). Rates of diffusional exchange between small cells and a measuring patch pipette. *Pflugers Arch.* 411 204–211. 10.1007/BF00582316 2451806

[B48] Shupliakov O. Low P. Grabs D. Gad H. Chen H. David C. (1997). Synaptic vesicle endocytosis impaired by disruption of dynamin-SH3 domain interactions. *Science* 276 259–263. 10.1126/science.276.5310.259 9092476

[B49] Smith C. Moser T. Xu T. Neher E. (1998). Cytosolic Ca2+ acts by two separate pathways to modulate the supply of release-competent vesicles in chromaffin cells. *Neuron* 20 1243–1253. 10.1016/S0896-6273(00)80504-8 9655511

[B50] Smith C. Neher E. (1997). Multiple forms of endocytosis in bovine adrenal chromaffin cells. *J. Cell Biol.* 139 885–894. 10.1083/jcb.139.4.885 9362507 PMC2139962

[B51] Sorensen J. B. (2004). Formation, stabilisation and fusion of the readily releasable pool of secretory vesicles. *Pflugers Arch.* 448 347–362. 10.1007/s00424-004-1247-8 14997396

[B52] Taraska J. W. Perrais D. Ohara-Imaizumi M. Nagamatsu S. Almers W. (2003). Secretory granules are recaptured largely intact after stimulated exocytosis in cultured endocrine cells. *Proc. Natl. Acad. Sci. U.S.A.* 100 2070–2075. 10.1073/pnas.0337526100 12538853 PMC149960

[B53] Van Hook M. J. Parmelee C. M. Chen M. Cork K. M. Curto C. Thoreson W. B. (2014). Calmodulin enhances ribbon replenishment and shapes filtering of synaptic transmission by cone photoreceptors. *J. Gen. Physiol.* 144 357–378. 10.1085/jgp.201411229 25311636 PMC4210432

[B54] Voets T. (2000). Dissection of three Ca2+-dependent steps leading to secretion in chromaffin cells from mouse adrenal slices. *Neuron* 28 537–545. 10.1016/S0896-6273(00)00131-8 11144362

[B55] Voets T. Neher E. Moser T. (1999). Mechanisms underlying phasic and sustained secretion in chromaffin cells from mouse adrenal slices. *Neuron* 23 607–615. 10.1016/S0896-6273(00)80812-0 10433271

[B56] von Gersdorff H. Matthews G. (1997). Depletion and replenishment of vesicle pools at a ribbon-type synaptic terminal. *J. Neurosci.* 17 1919–1927. 9045721 10.1523/JNEUROSCI.17-06-01919.1997 PMC6793761

[B57] von Ruden L. Neher E. (1993). A Ca-dependent early step in the release of catecholamines from adrenal chromaffin cells. *Science* 262 1061–1065. 10.1126/science.8235626 8235626

[B58] Wu L. G. Hamid E. Shin W. Chiang H. C. (2014). Exocytosis and endocytosis: modes, functions, and coupling mechanisms. *Annu. Rev. Physiol.* 76 301–331. 10.1146/annurev-physiol-021113-170305 24274740 PMC4880020

[B59] Wu X. S. Wu L. G. (2009). Rapid endocytosis does not recycle vesicles within the readily releasable pool. *J. Neurosci.* 29 11038–11042. 10.1523/JNEUROSCI.2367-09.2009 19726662 PMC2757152

[B60] Zhou Z. Misler S. (1995). Action potential-induced quantal secretion of catecholamines from rat adrenal chromaffin cells. *J. Biol. Chem.* 270 3498–3505. 10.1074/jbc.270.8.3498 7876083

