# Supplementary material for: Sustained Exocytosis after Action Potential-Like Stimulation at Low Frequencies in Mouse Chromaffin Cells Depends on a Dynamin-Dependent Fast Endocytotic Process
Source: Front Cell Neurosci. 2016 Jul 26;10:184. doi: 10.3389/fncel.2016.00184 (PMC4960491; doi:10.3389/fncel.2016.00184)
Supplement: Supplementary file 1 [file Image_1.PDF]

Figure S1

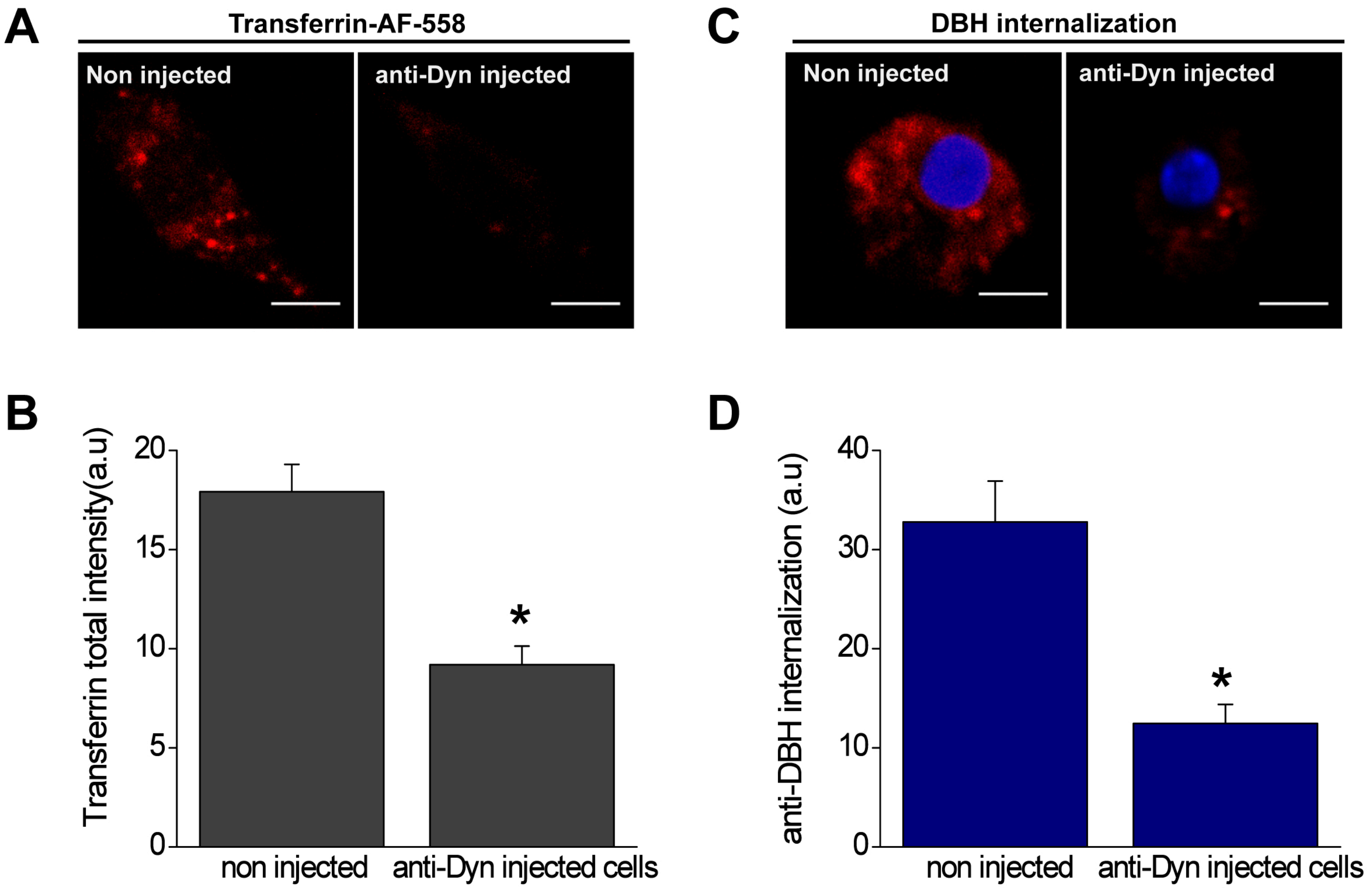

Microinjection of the anti-dynamin antibody impairs both constitutive and compensatory endocytosis in mouse chromaffin cells. Cultured chromaffin cells were injected with 7 nM of the anti-dynamin antibody (anti-Dyn injected, BD Biosciences) by using an InyectMan NI2 system (Eppendorf) and glass micropipettes with an internal tip diameter of 0.5 µm (Femtotips, Eppendorf); injection time and pressure were 0.2 s and 125 hPa, respectively. To avoid changes in pH chromaffin cells were kept in DMEM-F12 without HCO_3 during microinjection. **(A-B)** The effect of anti-Dyn injection on constitutive endocytosis was determined by using a transferrin uptake assay. Briefly, cells were incubated during 10 min at 4°C in a Hepes-based solution (mM: 135 NaCl, 5 KCl, 1 $CaCl_2$, 1 Mg Cl_2, 10 Hepes-NaOH, 20 glucose, 20% bovine-serum-albumin, pH 7.4) after injection and then incubated in the presence of 0.1 µM of the transferrin-conjugate Transferrin-AF-558 (Invitrogen) at 37°C during 5 min. Subsequently, cells were fixed with 4% p-formaldehyde (PFA) during 15 min and visualized by confocal microscopy (upright Eclipse Nikon 80i) using an immersion-oil 100x magnification objective and identical exposure settings between compared samples. Non injected cells in the same plate were used as control. **(A)** Representative images of the internalized Transferrin-AF-558 in a non-injected (left panel) and an anti-Dyn injected (right panel) cell. Scale bar= 5 µm. **(B)** Transferrin uptake was estimated by measuring the total intensity fluorescence of Transferrin-AF-558 inside the cell. Bars correspond to means ± SEM of transferrin uptake in cells non-injected (n=13) or injected with anti-Dyn (n=16); *$p<0.05$ (Unpaired t-test). Note that injection with anti-Dyn significantly reduced the transferrin uptake in mouse chromaffin cells**.** **(C-D)** The effect of anti-Dyn injection on compensatory endocytosis was determined by using a dopamine-beta hydroxylase (DBH) internalization assay. Briefly, after injection, cells were stimulated with 100 mM KCl for 1 min to induce exocytosis, and then incubated in a Krebs-Hepes buffer (mM: 140 NaCl, 5.9 KCl, 2 $CaCl_2$, 1.2 Mg Cl_2, 10 Hepes-NaOH, pH 7.4) in the presence of a rabbit polyclonal antibody directed against DBH (Abcam) for 1 h at 4°C. Internalization of the anti-DBH antibody was allowed at 37°C during 10 min; after that, cells were fixed in 4% PFA during 15 min, permeabilized during 5 min with 0.1% triton X-100, developed with a secondary anti-rabbit Cy3-conjugated antibody, incubated with DAPI to stain nuclei and visualized by confocal microscopy using an immersion-oil 100x magnification objective and identical exposure settings between compared samples. Non injected cells in the same plate were used as control. **(C)** Representative images of DBH internalization in a non-injected (left panel) and an anti-Dyn injected (right panel) cell. Scale bar= 5 µm. **(D)** Compensatory endocytosis was estimated by measuring the total intensity fluorescence of the DBH staining inside the cells. Bars correspond to means ± SEM of DBH internalization in cells non-injected (n=11) or injected with anti-Dyn (n=10);*$p<0.05$ (Unpaired t-test). Note that injection with the anti-Dyn antibody significantly reduced DBH internalization in mouse chromaffin cells.

Figure S2

SUSTAINED EXOCYTOSIS AFTER ACTION POTENTIAL-LIKE STIMULATION IN MOUSE CHROMAFFIN CELLS DEPENDS ON A FAST ENDOCYTOTIC PROCESS. José Moya-Díaz, Yanina D. Álvarez, Mauricio Montenegro, Lucas Bayonés, Ana Verónica Belingheri, Arlek M. González-Jamett, Ana M. Cárdenas, and Fernando D. Marengo.

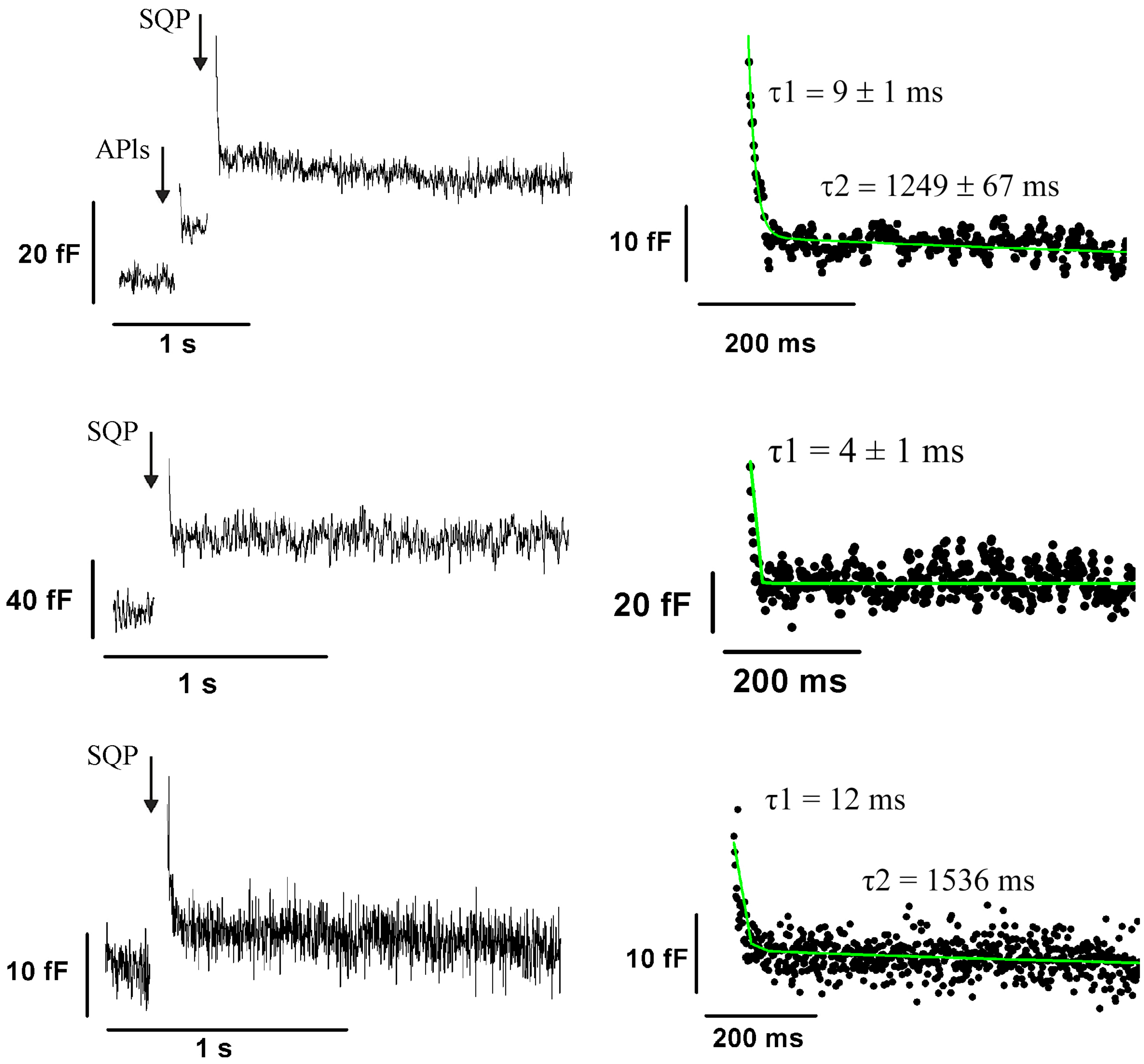

Immediately after the application of a depolarization pulse,many cells exhibited a brief capacitance transient. The left panels show examples of these capacitance transients after square 50 ms depolarizations (SQP), or after AP_{ls} (the first pulse in the upper panel). The right panels represent the decay of the same fast transients after SQP, fitted to single exponential functions. These capacitance transient became negligible 50 ms after the end of depolarization (exponential time constant = 14±2 ms; 14 measurements in 9 cells). For square depolarizations, after the fast transient it was common to observe a slow decay in capacitance ($\tau > 1$ s), probably associated to endocytosis. For AP_{ls}, the transient was followed by fast endocytosis ($\tau > 0.5$ s). To avoid any influence of this fast capacitance transient in our measurements, in all our records we did not consider the first 60 ms after the end of the depolarization pulse.

SQP: square 50 ms depolarization pulse; APls: action potential like stimulus. The figures on the right are fittings of the capacitance records induced by SQP, which are represented on the left.

Figure S3

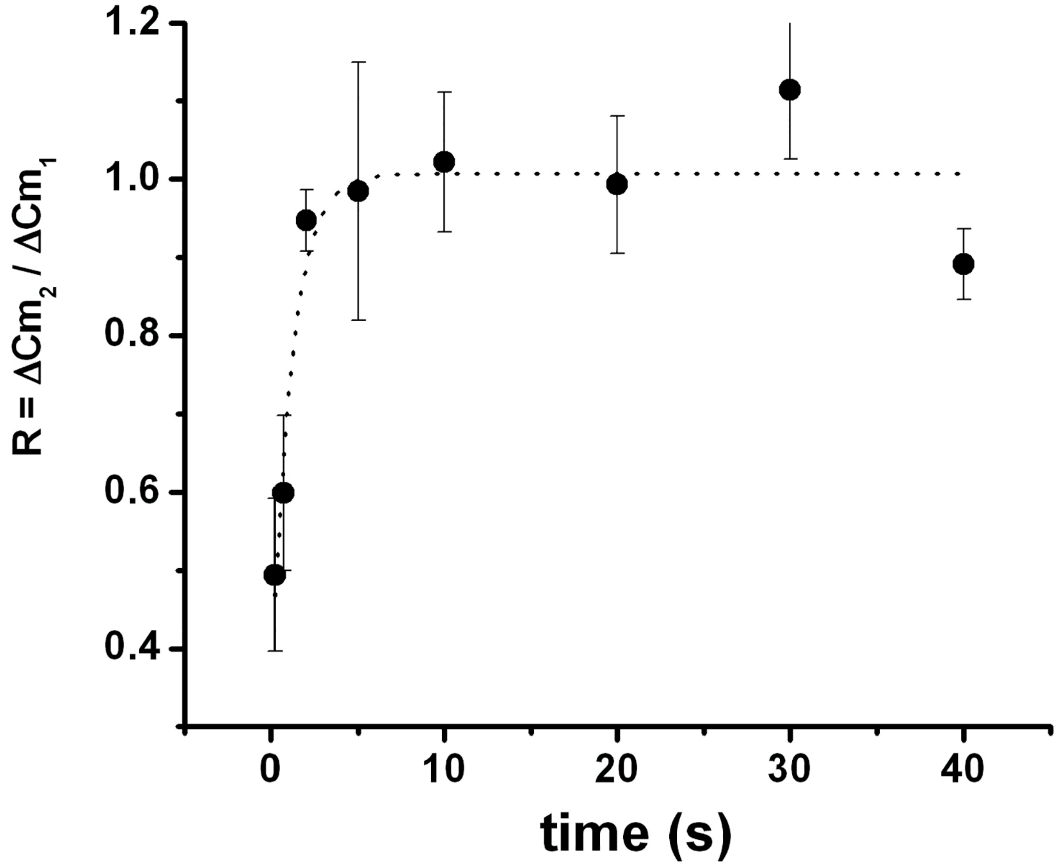

The relative replenishment of ETAP (expressed as $\Delta C_{m2}/\Delta C_{m1}$; where ΔC_{m1} and ΔC_{m2} represents the change in capacitance induced by the first and the second AP_{ls}, respectively) was plotted against the time interval between the pairs of AP_{ls}. This graph was obtained from a set of data, which are independent of that presented in Fig 3. Here, we analyzed the effect of long times between stimuli to rule out the possibility that the fast recovery is a transient process that finally decays after prolong periods of time. The present results reject that possibility. The averaged values were fitted to a monoexponential growing function ($R = R_O + A.(1 - e^{\frac{-t}{\tau}})$), obtaining a value at time zero Ro of 0.36 ± 0.12, an asymptote A of 0.65 ± 0.12, a time constant τ of 1.10 ± 0.48 seconds, and a correlation coefficient R > 0.94954.

Figure S4

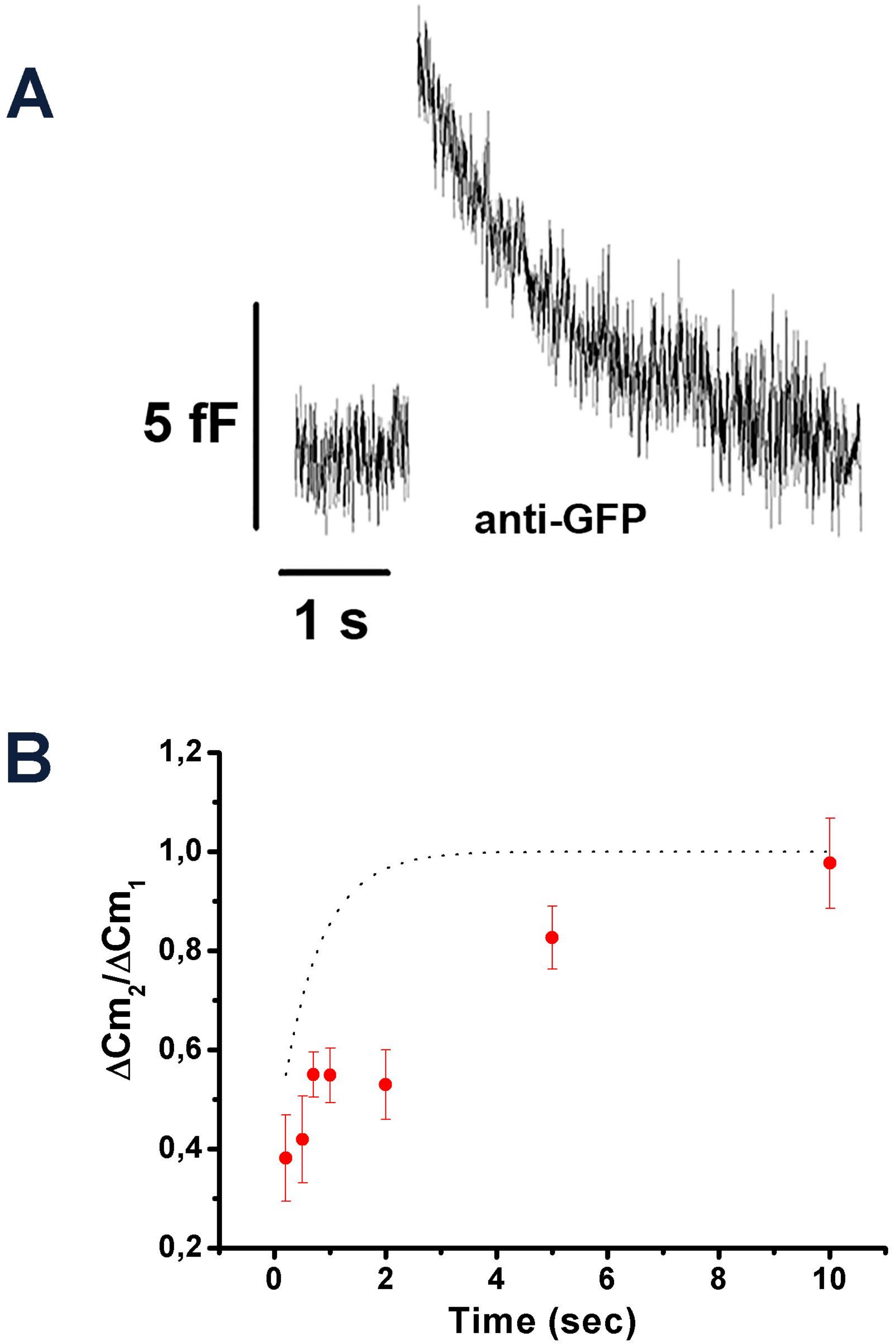

A: This figure represents the averaged C_m decay associated to endocytosis (n=5) in cells dialyzed with an anti-GFP antibody, which was added to the internal solution at the concentration used for anti-Dyn experiments (7 nM). B: Replenishment of ETAP in presence of an anti-dynamin antibody (14 nM) added to the internal solution (n = 8). For comparison, the fitted curve obtained in control conditions (taken from Fig. 3B) is shown as dotted line.
